# Mongolian medicine Wulanwendusu-11 alleviates myocardial ischemia-reperfusion injury by modulating the intestinal microbiota and associated metabolic pathways

**DOI:** 10.3389/fmicb.2025.1693472

**Published:** 2026-01-09

**Authors:** Wuyunsiqin, Tingting Bai, Dandan Yang, Hashentuya, Namila, Chelegeer Jin, Tana, Pengwei Zhao, Minjie Wang, Menggenduxi

**Affiliations:** 1 School of Traditional Mongolian Medicine, Inner Mongolia Medical University, Hohhot, China; 2 Key Laboratory of Quality Research and Pharmacodynamic Evaluation of Traditional Chinese Medicine and Mongolia Medicine, Inner Mongolia Medical University, Hohhot, China; 3 School of Basic Medicine, Inner Mongolia Medical University, Hohhot, China

**Keywords:** myocardial ischemia-reperfusion injury, gut microbiota, Wulanwendusu-11, metagenomics, untargeted metabolomics

## Abstract

**Objective:**

Wulanwendusu-11 (WLWDS-11) is a commonly used Mongolian medicine for treating cardiovascular diseases. However, its regulating effect on intestinal flora-host metabolism in relieving chronic myocardial ischemia-reperfusion injury (MIRI) is still unclear. Therefore, this study aims to systematically explore the cardioprotective mechanism of WLWDS-11 from the perspective of metabolic interaction between intestinal microbiota and host.

**Methods:**

C57BL/6J mice were randomized into six experimental groups: MIRI model, sham surgery, and treatment groups for compound Danshen dripping pills (CDDP) plus three dosages of WLWDS-11 (denoted WLWDS-11-L, WLWDS-11-M, and WLWDS-11-H). General physiological indicators of mice in each group were observed, body weight, myocardial structure and pathological features were assessed by electrocardiogram, plasma cardiac enzyme levels. The cardiac function of mice was obtained by echocardiography. Immunohistochemical staining was used to detect the pathological changes in the heart. Immunofluorescence assay was used to detect the degree of apoptosis. Metabolomics and metagenomics were used to analyze treatment effects on intestinal microbiota and metabolites. Integrated analysis of the enriched oxidative phosphorylation and necrosis and apoptosis pathways. qRT-PCR and western blot were used to detect the expression of COX4I1, NDUFB8, SDHA, TFAM, RIPK1, RIPK3, MLKL and TNF-α.

**Results:**

WLWDS-11 (especially in high dose) can significantly improve the cardiac function, reduce the area of myocardial infarction and weaken apoptosis and fibrosis in MIRI mice. Metabolomic profiling revealed extensive metabolic alterations, pathway analysis implicated arginine/proline and unsaturated fatty acid metabolism, and hierarchical clustering identified specific correlations between differential flora (e.g., Kosakonia, Helicobacter spp.) and key metabolites. Integrated multi-omics analysis demonstrated that MIRI induces gut microbiota dysbiosis and systemic metabolic disturbances, characterized by the accumulation of oxidized lipids/lysophospholipids and disruption of critical metabolic pathways. The intervention of WLWDS-11 effectively reshaped the intestinal microbial community and made the metabolic spectrum return to normal. More importantly, correlation and network analysis confirmed the correlation between specific intestinal bacteria (such as Prevost, Kosakonia and Helicobacter) and host metabolites, and formed a flora-metabolite axis regulated by WLWDS-11. KEGG pathway analysis further confirmed the effects of the treatment on key pathways, including necrotizing apoptosis and oxidative phosphorylation. From the point of view of mechanism, WLWDS-11 reversed the mitochondrial dysfunction induced by MIRI by up-regulating the expressions of COX4I1, NDUFB8, SDHA and TFAM. By inhibiting the RIPK 1/RIPK 3/MLKL pathway and TNF-α, necrotizing apoptosis and inflammatory response are inhibited. These results suggest that WLWDS-11 may protect MIRI’s heart by regulating the metabolic pathway of flora.

**Conclusion:**

WLWDS-11 positively reshaped the gut microbial environment by suppressing pathogenic bacteria and promoting beneficial strains, thereby fostering eubiosis, attenuating cardiac pathology, and ultimately conferring cardio protection. These findings identify WLWDS-11 as a potential candidate drug and provide a molecular mechanistic basis for the clinical treatment of MIRI.

## 1 Introduction

Acute myocardial infarction (AMI) continues to be one of the leading causes of mortality and disability worldwide. Both the incidence and mortality rates of AMI have been rising annually. According to the Global Burden of Disease study, ischemic heart disease (IHD)—the pathological basis of AMI—remains the leading cause of death globally, though with notable regional variation (Yusof et al., 2025). As such, IHD represents a critical public health challenge in modern societies, posing severe threats to human health and generating substantial economic burdens (Levine et al., 2016). The China Cardiovascular Health and Disease Report 2022 further high lights this issue, reporting MI mortality rates of 78.65 and 60.29 per 100,000 population in rural and urban areas, respectively, alongside significant regional disparities in in-hospital mortality.

Current management strategies for ischemic heart disease (IHD) include percutaneous coronary intervention and coronary artery bypass grafting, often in conjunction with anticoagulant and antiplatelet therapies. These interventions are designed to promptly restore blood flow to ischemic myocardium (i.e., reperfusion) and have been shown to substantially mitigate the acute progression of myocardial infarction (MI) (Doenst et al., 2019; Lippi et al., 2013). However, the process of reperfusion itself can induce stress-related damage to cardiomyocytes—a phenomenon known as myocardial ischemia-reperfusion injury (MIRI)—which may become life-threatening in severe instances (Severino et al., 2020; Stone et al., 2023; Pagliaro and Penna, 2023). Consequently, the development of effective approaches to attenuate MIRI carries significant clinical implications and represents a major focus in contemporary cardiovascular research.

The intestinal system is the body’s largest digestive organ and one of its primary interfaces with the external environment. The human gastrointestinal tract hosts a vast community of microorganisms, collectively known as the gut microbiota, which includes bacteria, viruses, and fungi. Bacteria constitute the overwhelming majority, with an estimated population of 100 trillion (Kamada et al., 2013). Approximately 90% of these bacteria reside in the colon, where they play crucial immunomodulatory roles and significantly influence the development and function of the intestinal mucosal immune system (Kelly et al., 2005; Kamada and Núñez, 2014; James et al., 2020; Zhou et al., 2020). Extensive research has demonstrated that the gut microbiota and its metabolites are crucial in maintaining intestinal homeostasis, significantly impacting human health and influencing the development of various diseases (e.g., cardiovascular and metabolic disorders) (Chen et al., 2021). As such, in-depth investigation of microbially mediated disease mechanisms can provide new insights to support treatment and prevention.

Wulanwendusu-11 (WLWDS-11) is a traditional Mongolian medicine formulation with a long history of clinical use (Surongzabu, 2011). Its effects are regarded as warming the stomach, separating clear and turbid blood, nourishing the heart, and thinning the blood. It is traditionally used in cardiovascular diseases (CVD) such as coronary heart disease, shortness of breath, and hypertension. The principal herb in this formulation, Dan Shen (*Salvia miltiorrhiza*), has been shown to ameliorate MIRI through mechanisms involving the suppression of oxidative stress and preservation of mitochondrial function (Qiao and Xu, 2016; Zhuo et al., 2021; Hu et al., 2020; Hu et al., 2023). Nutmeg and jujube, two other components of the formula, also possess recognized efficacy in the management of cardiovascular diseases. Furthermore, sea buckthorn is a medicinal plant abundant in flavonoids, known for its antioxidant and anti-atherosclerotic properties (Pichiah et al., 2012; Olas, 2018). Notably, previous studies suggest that dietary flavonoids may contribute to the regulation of gut microbiota composition (Ferreira et al., 2020; Osborn et al., 2021). In turn, microbial-derived metabolites—including lipopolysaccharides (LPS), trimethylamine-N-oxide (TMAO), and short-chain fatty acids (SCFAs)—can systemically influence the cardiovascular system, modulating developmental and physiological processes, and ultimately affecting cardiac health (Desai et al., 2023).

As a classic traditional Chinese medicinal formula, compound Danshen preparations (CDDP) have been extensively investigated for their cardioprotective potential against MIRI. Preclinical studies have demonstrated that compound Danshen Tablets alleviate MIRI-induced ventricular remodeling by regulating the AMPK/mTOR signaling pathway, whereas pretreatment with CDDP enhances autophagic flux to mitigate myocardial MIRI. Furthermore, expert recommendations issued by the Chinese Geriatrics Society provide evidence-based support for the rational clinical application of CDDP, bridging the gap between preclinical research and clinical practice (Li et al., 2024; Liu et al., 2023; Writing Group of Recommendations of Expert Panel from Chinese Geriatrics Society on the Clinical Use of Compound Danshen Dripping Pills, 2017).

Given the established significance of gut microbiota in cardiovascular disease (CVD), along with the fact that WLWDS-11 is abundant in flavonoids, organic acids, and polysaccharides a compelling theoretical foundation exists for investigating this traditional formulation in mitigating myocardial ischemia-reperfusion injury (MIRI) through gut microbiota modulation. Accordingly, this study aimed to exploit the interplay between gut microbiota and the cardiovascular system to achieve therapeutic effects in a mice model of MIRI. By integrating metagenomics and broad-targeted metabolomics, we explored the potential mechanisms through which WLWDS-11 alleviates MIRI pathology via gut microbiota regulation. Our ultimate objective was to identify potentially relevant gut microbial taxa and metabolites associated with MIRI, so as to provide insights for subsequent in-depth investigations.

## 2 Materials and methods

### 2.1 Treatments and reagents

The WLWDS-11 used in this study was sourced from the Inner Mongolia Autonomous Region Hohhot Traditional Chinese Medicine and Mongolian Medicine Hospital (#Z100061), and contained individual plants in the following quantities: *S. miltiorrhiza* (195 g), *M. fragrans* (122 g), *Z. jujuba* (146 g), *C. cassia* (25 g), *K. galanga* L. (49 g), *C. sappan* (49 g), cassia seeds (61 g), *P. indicus* Willd. (98 g), *Inula helenium* L. (97 g), *R. Aucklandiae*. (97 g), and *Hippophae rhamnoides* L. (61 g). WLWDS-11 was prepared in accordance with the 2007 Edition of Inner Mongolia Mongolian Medicine Preparation Specifications, with key processes conducted in a Class D cleanroom (300,000-class). Briefly, 11 medicinal slices were depurated and reserved for use. Subsequently, the slices were precisely weighed under quality control, followed by rechecking and documentation. Separately, stable-component slices were sterilized by pulsating steam for 30 min, while volatile-component slices were spread thinly and subjected to UV sterilization for 30 min. Thereafter, the sterilized slices were co-pulverized into fine powder, which was passed through an 80-mesh sieve (≥95% of the powder passed through a 100 mesh sieve); 100 g samples were collected at the start, middle, and end stages to test for fineness. Finally, the powder was mixed in a three-dimensional mixer for 30 min, and samples were taken to confirm color uniformity. A representative UPLC chromatogram (Supplementary Figure S1) confirmed the presence of key markers (e.g., salvianolic acid B, tanshinone IIA). Other chemicals and reagents were purchased from commercial suppliers, including methanol, acetonitrile, ammonium hydroxide, 2-propanol, and sodium carboxymethylcellulose (CMC-Na) (all from CNW Technologies); ammonium acetate and acetic acid (both from Sigma-Aldrich); and compound Danshen dripping pills (CDDP; from Tasly Pharmaceutical Group Co., Ltd., China).

### 2.2 Instruments and equipment

The following instruments were used in this study: DM3000 laboratory microscope (Leica), LC-4012 low-speed centrifuge (Anhui USTC Zonkia Scientific Instruments Co., Ltd.), Precisa XJ620M electronic analytical balance (Shanghai Tianmei Balance Instrument Co., Ltd.), Excelsior ES fully automated tissue processor (Thermo Fisher Scientific), HistoStar embedding workstation (Thermo Fisher Scientific), Epredia HM 340E rotary microtome (Thermo Fisher Scientific), slimline hotplate (Thermo Fisher Scientific), and KH20R benchtop high-speed refrigerated centrifuge (Anhui USTC Zonkia Scientific Instruments Co., Ltd.).

### 2.3 Animals and treatment

The study protocols involving animals were reviewed and approved by the Animal Ethics Committee of Inner Mongolia Medical University (Permit No. YKD202503005), ensuring adherence to ethical principles and compliance with the NIH Guide for the Care and Use of Laboratory Animals and the ARRIVE guidelines. Eight-week-old male C57BL/6J mice were sourced from SPF (Beijing) Biotechnology Co., Ltd. [Certificate No. SYXK(Jing) 2024-0001]. The animals were acclimatized and housed in a specific pathogen-free (SPF) environment with controlled temperature (23 ± 1 °C), humidity (55 ± 5%), and a 12-h light/dark cycle. Standard rodent chow and water were provided ad libitum, and all enrichment and housing conditions were designed to meet their physiological needs. To minimize confounding variables, mice were housed separately by experimental group (6 animals per cage).

### 2.4 Animal modeling and grouping

After a one-week acclimatization period, 72 male C57BL/6J mice were assigned to six groups (*n* = 12 per group) using a simple randomization method. The specific procedure was as follows: a computer-generated random sequence (using the RAND function in Excel) was used to assign a unique random number to each mouse. Mice were sequentially allocated to each group based on the random number order or pre-defined grouping criteria, ensuring no subjective bias during the grouping process: a sham surgery group, a myocardial ischemia-reperfusion injury (MIRI) model group, and four treatment groups. The treatment groups received either CDDP (0.104 g/kg/day) or low-, medium-, or high-dose WLWDS-11 (0.39, 0.78, and 1.55 g/kg/day, respectively). All compounds were prepared as suspensions in 0.5% (w/v) sodium carboxymethyl cellulose (CMC-Na) and administered once daily by oral gavage for 4 weeks; the MIRI and sham groups received the vehicle (0.5% CMC-Na) only. Twelve hours after the final administration, the MIRI model was established.

During the surgery, investigators were blinded to group assignments. Mice were anesthetized with 2% isoflurane, intubated, and subjected to thoracotomy. The left anterior descending coronary artery was ligated for 30 min, followed by reperfusion. Sham-operated mice underwent the same surgical procedure without ligation. Postoperative monitoring continued for 7 days.

The medium dose of WLWDS-11 (0.78 g/kg/day) was derived from the maximum adult human daily dose (6 g/70 kg), converted using a standard factor of 9.01. The high and low doses were set as double and half of the medium dose, respectively. The CDDP dose was determined following a similar rationale.

A CONSORT-style flowchart (Supplementary Figure S2) outlines sample allocation, exclusion criteria, and final group sizes. For metabolomic and metagenomic analyses, 6 mice were randomly selected from each group (*n* = 12). This sample size was justified by a G*Power analysis (power = 0.8, *α* = 0.05, effect size = 1.2) and is consistent with common practice in omics studies. The remaining 6 mice per group were reserved for validating omics findings and mitigating potential sample loss, thereby optimizing resource utilization. Randomization and blinding were maintained throughout sampling, testing, and data analysis to prevent bias and ensure that omics samples were representative of each experimental group.

### 2.5 Electrocardiographic and echocardiographic examinations

Mice were placed in an induction chamber containing 2% isoflurane with continuous oxygen supply at 2 L/min for anesthesia induction, then connected to a small-animal electrocardiograph to monitor ST-segment changes at the following time points: before coronary ligation, 30 min after ligation, and more than 30 min after reperfusion. These electrocardiographic (ECG) changes served as key indicators for confirming the successful establishment of the myocardial ischemia-reperfusion injury (MIRI) model. Echocardiographic parameters were acquired using the Vinno6LAB imaging system (VINNO Technology, Suzhou, China). The mice were anesthetized with 2% isoflurane, M-mode echocardiography was utilized to measure left ventricular parameters, including ejection fraction (EF%), fractional shortening (FS%) and left ventricular internal dimension systole (LVIDs mm). Cardiac function was subsequently assessed by echocardiography on the seventh day after modeling (Supplementary Figure S3).

### 2.6 ELISA assay

Blood samples were collected from the posterior-orbital venous plexus, centrifuged at room temperature (15,000 × g, 20 min), and the supernatants removed. A second centrifugation step (15,000 × g, 20 min) was performed at 4 °C, then supernatants were removed and the samples were analyzed using commercial ELISA assay kits for serum creatine kinase (CK; R&D Systems, Inc., United States, #F00708), creatine kinase-MB isoenzyme (CK-MB; R&D Systems, Inc., United States, #F08185), lactate dehydrogenase (LDH; R&D Systems, Inc., United States; #F11858), cardiac troponin I (cTnI; R&D Systems, Inc., United States, #F12235), connective tissue growth factor (CTGF; R&D Systems, Inc., United States, #F03777), transforming growth factor beta-1‌ (TGF-β1; R&D Systems, Inc., United States, #F12041), and galectin-3 (Gal-3; R&D Systems, Inc., United States, #F05349). ATP detection in cardiac tissue was performed using cardiac homogenates (ATP; Beyotime Biotech, Inc., China, #S0026), following the manufacturer’s instructions in each case. A plate reader was used to measure the absorbance of each well at a wavelength of 450 nm.

### 2.7 Triphenyltetrazolium chloride staining

Mice were euthanized by anesthesia, then cardiac tissue for triphenyltetrazolium chloride (TTC) staining was excised and frozen, placed in covered Petri dishes, and stored at −20 °C for 30 min. Specimens were sliced into five pieces (1.5 mm thick) with a blade, transferred to Petri dishes, stained with 2% TTC, then incubated at 37 °C in the dark for 20 min. Following incubation, the staining solution was aspirated and fixative was added. Cardiac tissue for histopathologic staining was excised and fixed by placing in 10% neutral formaldehyde for 24 h. These specimens were further processed by gradient ethanol dehydration, xylene fixation, paraffin embedding, sectioning, staining with hematoxylin and eosin (Medical Discovery Leader Co., Ltd., China, #MD911477), and neutral gum sealing. Lesions were then observed by light microscopy.

### 2.8 TUNEL assay

Cardiac tissue samples were stained using a commercial TUNEL assay kit [Yeasen Biotechnology (Shanghai) Co., Ltd., China, #40306ES60]. Tissue sections underwent washing three times in PBS, with each wash lasting 5 min to remove residual fixatives and contaminants. Samples were fixed with 4% paraformaldehyde for 10–30 min, followed by a single PBS wash. Permeabilization was achieved using 0.1% Triton X-100 or 0.1% SDS for 5–10 min at room temperature. Subsequently, terminal deoxynucleotidyl transferase (TdT) enzyme and fluorescein-labeled dUTP were applied as instructed by the manufacturer’s guidelines. Samples underwent incubation at 37 °C for 2 h and then three PBS washes for 5 min each, before drying in the dark and being mounted with an anti-fluorescence quenching mounting medium. After coverslip placement, samples were examined under a fluorescence microscope at 400 × magnification to capture images. The sections were observed under a fluorescence microscope (Zeiss Microscope) at 400× magnification.

### 2.9 Detection of target molecule expression: quantitative real-time polymerase chain reaction and western blotting analysis

Total RNA was extracted from cardiac tissues using TRIZOLTRIzol reagent (Aidlab Biotechnologies Co., Ltd., China, #RN0102) according to the manufacturer’s instructions for subsequent quantitative real-time polymerase chain reaction (qRT-PCR) analysis. cDNA was synthesized from the purified RNA using the ExonGen reverse transcription kit (ExonScript RT SuperMix with dsDNase; ExonGen Inc., South Korea, #A502). Subsequently, qRT-PCR was performed on a LongGene Real-Time PCR System (Q2000B, LongGene Scientific Instruments (Shanghai) Co., Ltd., China) using SYBR Green Real-Time PCR Master Mix (ABI-Invitrogen, United States, #4472920). Relative mRNA expression levels were determined using the comparative Ct (2^−ΔΔCT^) method with β-actin mRNA as the reference gene. The mRNA-specific primers for COX4I1, NDUFB8, SDHA, TFAM, RIPK1, RIPK3, MLKL, and TNF-α and β-actin are listed in Table 1.

**Table 1:** Primers used in this study for RT-qPCR.

Target name	Primer	
β-actin	F	CTCCTGAGCGCAAGTACTCT
	R	TACTCCTGCTTGCTGATCCAC
	R	CACTGAAGGCTTCTTTGGGTCG
COX4I1	F	TCATTGGCTTCACTGCGCTCGT
	R	TCCAGCATTCGCTTGGTCTGCA
NDUFB8	F	CGCCAAGAAGTATAACATGCGAG
	R	CCTCTCATGCTGTGATCGGTTG
SDHA	F	GAGATACGCACCTGTTGCCAAG
	R	GGTAGACGTGATCTTTCTCAGGG
TFAM	F	GAGGCAAAGGATGATTCGGCTC
	R	CGAATCCTATCATCTTTAGCAAGC
RIPK3	F	GAAGACACGGCACTCCTTGGTA
	R	CTTGAGGCAGTAGTTCTTGGTGG
MLKL	F	CTGAGGGAACTGCTGGATAGAG
	R	CGAGGAAACTGGAGCTGCTGAT
RIPK1	F	GACTGTGTACCCTTACCTCCGA
	R	CACTGCGATCATTCTCGTCCTG
TNF-α	F	GGTGCCTATGTCTCAGCCTCTT
	R	GCCATAGAACTGATGAGAGGGAG

About 20 mg of cardiac tissue was taken on ice and homogenized in RIPA lysis buffer (Beyotime Biotechnology Co., Ltd., Shanghai, China, #P0013C) containing protease inhibitor cocktail (Beyotime Biotechnology Co., Ltd., Shanghai, China, #P1005) using a pre-cooled grinder or ultrasonic cell disruptor (XC-CD, Xianchang Technology Co., Ltd., China) under ice bath. After centrifugation at 12,000 rpm for 10 min at 4 °C, the supernatant was collected into a clean tube. Protein concentration was determined by the BCA method using a BCA Protein Assay Kit (MDL Biotechnology Co., Ltd., China, #MD913053). Samples were adjusted to the same concentration by adding an appropriate volume of loading buffer and deionized water, then heated at 100 °C for 10 min to denature proteins. After cooling on ice, the denatured protein samples were stored at −20 °C.

Equal amounts of total proteins were subjected to SDS-PAGE electrophoresis using precast gel kits (MDL Biotechnology Co., Ltd., China, #MD911919) or self-prepared gels (10% separating gel and 5% stacking gel). Protein separation was carried out using sodium dodecyl sulfate-polyacrylamide gel electrophoresis (SDS-PAGE) with 20 μg of protein loaded per well. The separated proteins were then transferred to a nitrocellulose membrane (GVS S.p.A., United States, #ISEQ00010). The membrane was sealed using a 3% non-fat milk solution. The primary antibodies including anti-NDUFB8 (#83216-3-RR, Proteintech Group, Inc., Wuhan, China), anti-MLKL (Proteintech, Wuhan, China, #66675-1-Ig), and anti-p-MLKL (Proteintech Group, Inc., Wuhan, China, #66675-1-Ig) were applied overnight at 4 °C. Following incubation with secondary antibodies HRP-conjugated Goat anti-Mouse IgG (Saiwei Biotechnology Co., Ltd., Shanghai, China, #C030205), HRP-conjugated Goat anti-Rabbit IgG (Saiwei Biotechnology Co., Ltd., Shanghai, China, #C030212), or HRP-conjugated Rabbit anti-Rat IgG (Saiwei Biotechnology Co., Ltd., Shanghai, China, #C030226) for 1 h at room temperature, protein bands were detected using chemiluminescent reagents and a chemiluminescent imaging system (ChemiScope6100, CLINX Science Instruments Co., Ltd., Shanghai, China).

### 2.10 Metabolite analysis

Metabolites were extracted from 50 μL serum using 200 μL of methanol/acetonitrile (1:1) containing internal standards on a Starlid^™^ workstation. After shaking (750 rpm, 5 min) and standing (5 min), samples were filtered through a protein precipitation plate (Manhage #H3006079). Quality control (QC) samples were prepared by pooling equal volumes of filtrates from each experimental group, and QC measures included repeated injections, retention time stability, and internal standard normalization. Chromatographic separation was performed on a Vanquish UHPLC system with a Waters ACQUITY UPLC BEH Amide column (2.1 × 50 mm, 1.7 μm). The mobile phase consisted of 25 mM NH₄OAc/NH₄OH in water (A) and acetonitrile (B). The injection volume was 2 μL with samples maintained at 4 °C. LC-MS/MS analysis was carried out using an Orbitrap Exploris 120 mass spectrometer. Key parameters included: sheath gas 50 Arb, auxiliary gas 15 Arb, capillary temperature 320 °C, full MS resolution 60,000, MS/MS resolution 15,000, stepped collision energy 20/30/40 eV, and spray voltage ±3.8 kV (positive) or −3.4 kV (negative).

### 2.11 Metagenomic sequencing

Total microbial DNA was extracted using the MagBeads Fast DNA Kit for Soil, quantified by Qubit Fluorometer, and assessed via agarose gel electrophoresis. Sequencing libraries (400 bp insert) were prepared with the TruSeq DNA Nano Kit and sequenced on an Illumina NovaSeq platform (PE150). Raw reads were processed by removing adapters (Cutadapt) and filtering low-quality sequences (fastp, *Q* < 20). Host-derived reads were excluded by alignment to the *Mus musculus* genome (Bowtie2, identity ≥95%, coverage ≥90%). Contigs were assembled with MEGAHIT (meta-large mode), clustered with MMseqs2 (identity ≥95%, coverage ≥90%), and taxonomically classified against the NCBI nucleotide database. Contigs assigned to Viridiplantae or Metazoa were discarded. Gene prediction was performed with MetaGeneMark, and CDSs were clustered (MMseqs2, identity ≥90%, coverage ≥90%). Gene abundance was quantified with Salmon (--meta –minScoreFraction = 0.55) and normalized as TPM. Functional annotation was carried out against KEGG, eggNOG, and CAZy databases using MMseqs2. GO terms were derived via eggNOG-mapper and slimmed using map2slim, while KEGG orthology annotations were obtained from KOBAS.

### 2.12 Statistical analysis

Data are presented as mean ± standard error of the mean (SEM). For multiple comparisons, one-way ANOVA with Tukey’s *post-hoc* test was used, and *p*-values were adjusted using the Benjamini–Hochberg FDR method. All statistical analysis. A value of *p* < 0.05 was considered as statistical difference.

## 3 Results

### 3.1 WLWDS-11 ameliorates cardiac injury in MIRI mice

**Figure 1:**
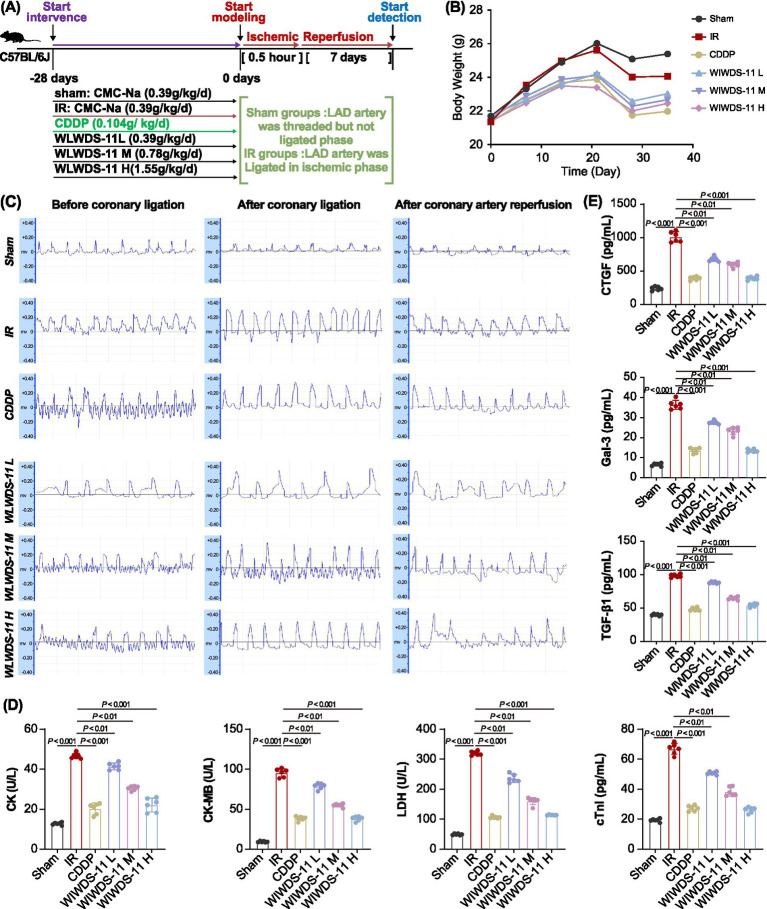
WLWDS-11 ameliorates cardiac injury in MIRI mice: evaluation based on key detection indices (physiology, electrophysiology, myocardial injury, fibrosis). **(A)** The experimental flow chart (including modeling time, administration time, etc.). **(B)** Statistical results of the body weight of mice in each group. **(C)** Electrocardiographic (ECG) changes in mice before coronary artery ligation, after ligation, and after reperfusion in each group. **(D)** Comparison of serum myocardial injury marker levels (CK-MB, LDH, CK, cTnI) among different groups. **(E)** Result of serum levels of fibrosis-related cytokines (CTGF, Gal-3, TGF-β1) in each group (*n* = 6, mean ± SEM).

A schematic of the experimental workflow is shown in Figures 1A,B, along with body weight measurements for each group. Sham mice remained healthy with normal coat quality, responsiveness, appetite, and steady weight gain. MIRI mice exhibited significant weight loss, lethargy, dull fur, cold skin, reduced activity/appetite, and diarrhea. WLWDS-11-H mice showed slightly improved alertness and skin temperature but similar coat/appetite deficits and weight trends to MIRI mice. No mortality occurred.

In ECG tests, MIRI mice showed significantly higher heart rate compared with the sham group, along with ST segment elevation, indicating successful model establishment. Compared with MIRI mice, the heart rate and ST segment amplitude in the WLWDS-11-H group decreased significantly (Figure 1C).

ELISA assay showed that compared with sham mice, the MIRI model group showed significantly elevated total creatine kinase (CK), creatine kinase MB isoenzyme (CK-MB), lactate dehydrogenase (LDH), and cardiac troponin I (cTnI; all *p* < 0.001). In the WLWDS-11-H group, the levels of all four enzymes in WLWDS-11-H group were significantly decreased (all *p* < 0.001; Figure 1D). Serum measurements of myocardial fibrosis markers revealed that levels of TGF-β1, CTGF, and galectin-3 (Gal-3) were significantly elevated in MIRI model versus sham mice (all *p* < 0.001); however, the WLWDS-11-H group showed a significant decrease in serum levels of TGF-β1, CTGF, and Gal-3 relative to model mice (all *p* < 0.001; Figure 1E).

### 3.2 WLWDS-11 attenuates cardiac remodeling myocardial and apoptosis in MIRI mice

In the MIRI model group, multifocal myocardial necrosis was observed, with subsequent replacement by proliferating fibrous connective tissue (i.e., fibrosis). Interstitial collagen fiber deposition was evident, accompanied by prominent neovascularization and occasional punctate hemorrhages. Mild inflammatory cell infiltration was also present. Additionally, extensive myocardial atrophy was noted, characterized by reduced cellular volume and widened intercellular spaces.

**Figure 2:**
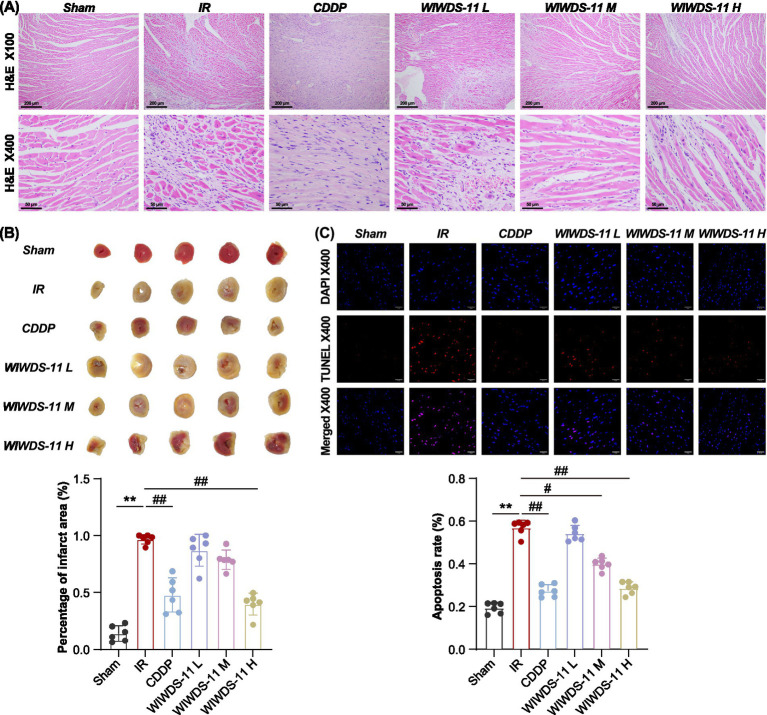
WLWDS-11 attenuates cardiac remodeling myocardial and apoptosis in MIRI mice. **(A)** Shows the results of HE pathological staining in each group, scale bar, 200 μm and 50 μm (*n* = 6, mean ± SEM). **(B)** Results and statistics of TTC staining. **(C)** TUNEL staining situation and result statistics, scale bar, 50 μm (*n* = 6, mean ± SEM).

Histopathological evaluation revealed distinct patterns of myocardial repair and injury across treatment groups (Figure 2A). In the CDDP group, small necrotic foci were superseded by fibrous connective tissue, accompanied by interstitial fibrosis and scattered inflammatory cell infiltrates. The WLWDS-11-L group exhibited replacement of large necrotic areas by myocardial fibrosis, along with interstitial collagen deposition, modest neovascularization, and numerous inflammatory infiltrates; myocardial atrophy was relatively limited, though characterized by reduced cell volume and widened intercellular spaces. In WLWDS-11-M mice, patchy necrosis was replaced by myocardial fibrosis, with interstitial collagen proliferation and only occasional punctate inflammatory cell infiltration; a small number of atrophied cardiomyocytes displayed reduced volume and widened interstices. Finally, WLWDS-11-H hearts showed that focal necrosis had been replaced by fibrous connective tissue, with interstitial fibrosis and occasional punctate inflammatory infiltrates; findings also included slight cardiomyocyte atrophy, reduced cell volume, and widened intercellular spaces.

The results of triphenyltetrazolium chloride (TTC) staining showed that, unlike sham mice, the MIRI model group exhibited obvious signs of MI, whereas WLWDS-11-H treatment had significantly decreased the extent of myocardial ischemia (Figure 2B).

As shown in Figure 2C, TUNEL staining revealed only a few scattered weakly positive cells in cardiac tissue from sham mice, with intact nuclear morphology. However, a large number of positive myocardial cells were observed in MIRI mice, displaying marked nuclear condensation and fragmentation and forming sheet-like aggregates; thus, the proportion of positive cells was significantly higher than for the sham group (*p* < 0.01). Interestingly, the intensity of positive staining and percentage of positive cells were lower in the CDDP and WLWDS-11-H groups (both *p* < 0.01), suggesting that the latter treatment in particular can effectively inhibit MIRI-induced apoptosis, limit the loss of viable cells, and attenuate cardiac injury.

### 3.3 WLWDS-11 ameliorates serum metabolic changes in MIRI mice

**Figure 3:**
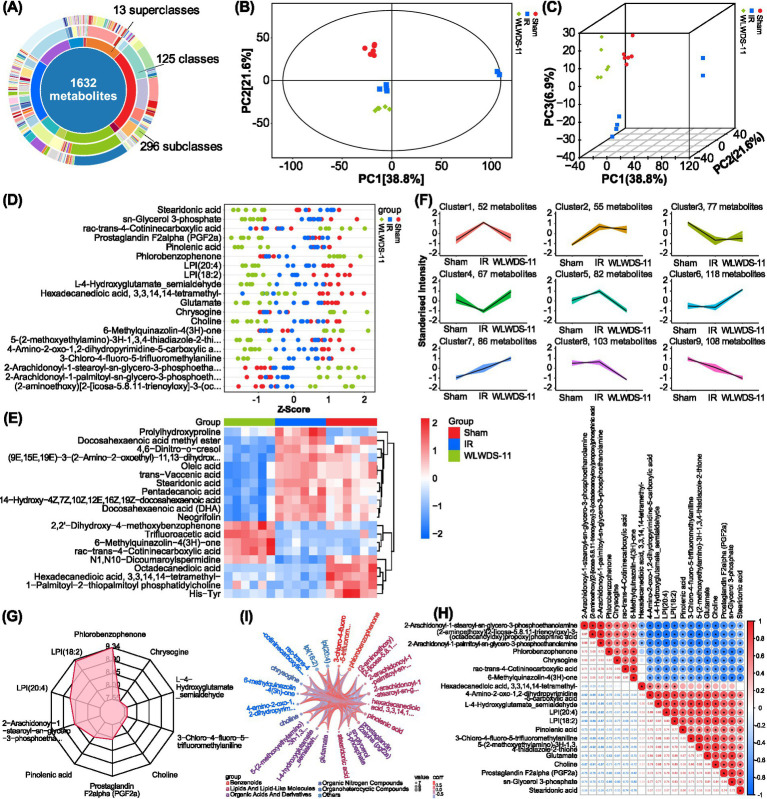
Metabolomic profiling reveals WLWDS-11-regulated metabolic perturbations and differential metabolite clustering in MIRI mice. **(A)** Donut chart of metabolite classification. **(B)** Dispersion point plot of PCA of all samples. **(C)** Three-dimensional dispersion point map of the PCA model of each group. **(D)** *Z*-score of each group. **(E)** Heat map of hierarchical cluster analysis of each group. **(F)** Is the *k*-means plot for all groups. **(G)** Radar chart of each group. **(H)** Correlation analysis heat map of each group. **(I)** Chord diagram of each group. ^*^p < 0.05.

Raw data were acquired for QC samples and 18 experimental samples, from which 38,292 peaks were extracted. After preprocessing as described above, 28,319 features were retained, including 1,632 qualitative secondary metabolites, 13 annotated secondary metabolites, 125 annotated tertiary metabolites, and 296 annotated quaternary metabolites. Substance classification details are summarized in Figure 3A. The horizontal axis PC[1] and vertical axis PC[2] in Figures 3B,C represent the scores of the first and second principal components, respectively, in principal component analysis (PCA) of metabolite profiles, noting that the samples fall primarily within the 95% confidence interval. The closer the distribution of sample points, the more similar the types and content of metabolites in those samples; conversely, the further apart the sample points, the greater the differences in metabolite profile between them. Observing the PCA plot of all samples allows trends in overall distribution to be readily visualized. The results indicated significant differences in overall metabolite profiles. Differential metabolites (DMs) were identified based on univariate and multivariate analyses (Table 2). The *Z*-score plot (Figure 3D) normalizes DMs, shown on the *y*-axis, across different samples using calculated *Z*-score values, shown on the *x*-axis. Different colors indicate samples from different groups, providing a visual representation of the distribution of DMs across experimental conditions. As such, this analysis allowed screening out of DMs, which often exhibit biological similarity and/or complementarity in terms of function, or are positively/negatively regulated by the same metabolic pathway, thereby resulting in similar or opposite occurrence patterns between groups. Hierarchical cluster analysis was performed on the top 20 significant DMs (each pairwise comparison) (Figure 3E), which proved similar between the sham and WLWDS-11-H groups, indicating that the treatment may improve intestinal metabolism in MIRI mice and further influence myocardial function. To investigate changes in metabolite profile across groups, the relative contents of all identified DMs were standardized via *Z*-score (based on the same screening criteria as above), followed by *k*-means clustering analysis. The results are shown in Figure 3F, where the *x*-axis indicates the sample group names and the *y*-axis represents the standardized relative content of metabolites. Clusters represent groups of metabolites with similar trends. Furthermore, radar chart analysis was performed on all DMs and the top 10 significantly different substances were selected (Figure 3G). We conducted Spearman’s correlation analysis to assess the pairwise relationships among differential metabolites (DMs) from different sources (Figure 3H). The correlation coefficient (*r*) ranges from −1 to +1, where the sign indicates the direction of the relationship and its absolute value denotes the strength. A chord diagram displays the resulting correlation network for the top 20 significant DMs (Figure 3I).

**Table 2:** Statistical table of differential metabolites.

Peak No.	Name	Inchikey	MS2 score	*m*/*z*	Formula	Level	Adduct	Library	RT time	ANOVA *p*-value	*q*-value
1	Fructose 6-phosphate	BGWGXPAPYGQALX-ARQDHWQXSA-N	3.84	259.02	C_6_H_13_O_9_P	Level1	[M − H]^−^	NGM_indatabase	270.80	0.00019904456744697	0.0038189316433135
2	Fructose 1-phosphate	ZKLLSNQJRLJIGT-UYFOZJQFSA-N	3.84	259.02	C_6_H_13_O_9_P	Level1	[M − H]^−^	NGM_indatabase	270.80	0.00019904456744697	0.0038189316433135
3	Mannose 1-phosphate	HXXFSFRBOHSIMQ-QTVWNMPRSA-N	3.83	259.02	C_6_H_13_O_9_P	Level1	[M − H]^−^	NGM_indatabase	270.80	0.00019904456744697	0.0038189316433135
4	9-Oxo-10(E),12(E)-octadecadienoic acid	LUZSWWYKKLTDHU-SIGMCMEVSA-N	3.93	293.21	C_18_H_30_O_3_	Level1	[M − H]^−^	NGM_indatabase	25.20	0.000184824621875907	0.00365506177856412
5	Tolmetin_sodium	UPSPUYADGBWSHF-UHFFFAOYSA-N	2.56	280.09	C_15_H_14_NNaO_3_	Level2	[M + H]^+^	NGM_pbdatabase	229.40	0.000184824621875907	0.00365506177856412
6	1-Myristoyl-sn-glycero-3-phosphocholine (LPC(14:0/0:0))	VXUOFDJKYGDUJI-OAQYLSRUSA-N	3.89	468.30	C_22_H_46_NO_7_P	Level1	[M + H]^+^	NGM_indatabase	124.70	0.000181179716630957	0.00359805637817116
7	Gibberellin_A61	JKQCGVABHHQYKQ-UHFFFAOYSA-N	2.22	333.17	C_19_H_24_O_5_	Level2	[M + H]^+^	NGM_pbdatabase	135.70	0.000177362305667106	0.00355213800154652
8	Nanaomycin E	SVGOJJZXRJJDLY-UHFFFAOYSA-N	2.66	317.06	C_16_H_14_O_7_	Level2	[M − H]^−^	NGM_pbdatabase	59.50	0.000170026949281366	0.00345656365879325
9	(Phenoxymethyl)penicilloic acid	FJTUHYOOCYRLJI-UHFFFAOYSA-N	2.21	369.11	C_16_H_20_N_2_O_6_S	Level2	[M + H]^+^	NGM_pbdatabase	156.80	0.000165611288187948	0.00338868935707695
10	1,2-Dilinoleoyl-sn-glycero-3-phosphoethanolamine	SSCDRSKJTAQNNB-DWEQTYCFSA-N	2.70	740.52	C_41_H_74_NO_8_P	Level2	[M + H]^+^	NGM_pbdatabase	38.40	0.000160334683917298	0.00331182925882856
11	Leu-Trp	BQVUABVGYYSDCJ-UHFFFAOYSA-N	2.68	318.18	C_17_H_23_N_3_O_3_	Level2	[M + H]^+^	NGM_pbdatabase	100.90	0.000150709825204158	0.00316613615723779
12	Batatasin III	VYQXIUVIYICVCM-UHFFFAOYSA-N	2.71	243.10	C_15_H_16_O_3_	Level2	[M − H]^−^	NGM_pbdatabase	31.90	0.000150670359599482	0.00316613615723779
13	1-O-Hexadecyl-sn-glycero-3-phosphocholine (LPC(O-16:0/0:0))	VLBPIWYTPAXCFJ-XMMPIXPASA-N	3.86	482.36	C_24_H_52_NO_6_P	Level1	[M + H]^+^	NGM_indatabase	124.70	0.000145479503113613	0.00308140168188062
14	4-(1-Pyrazolyl)benzaldehyde	PPGRDLZPSDHBIC-UHFFFAOYSA-N	2.75	173.07	C_10_H_8_N_2_O	Level2	[M + H]^+^	NGM_pbdatabase	24.80	0.00014467111351532	0.00306886986040476
15	GKK 1032B	GAPPHVJLWSRLAC-UHFFFAOYSA-N	2.58	500.27	C_32_H_39_NO_4_	Level2	[M − H]^−^	NGM_pbdatabase	119.70	0.000143453451699323	0.00305447992381437
16	Sph(d18:0)	OTKJDMGTUTTYMP-ZWKOTPCHSA-N	3.90	302.30	C_18_H_39_NO_2_	Level1	[M + H]^+^	NGM_indatabase	40.60	0.000140104663399818	0.00301033684584177
17	7-tert-Butyl-5,6,7,8-tetrahydro[1]benzothieno[2,3-d]pyrimidine-2,4(1H,3H)-dithione	UUJWCOHAPCQDIT-UHFFFAOYSA-N	2.62	309.05	C_14_H_18_N_2_S_3_	Level2	[M − H]^−^	NGM_pbdatabase	156.80	0.000135477809130918	0.00293600268663727
18	Pseudouridine	PTJWIQPHWPFNBW-GBNDHIKLSA-N	3.92	243.06	C_9_H_12_N_2_O_6_	Level1	[M − H]^−^	NGM_indatabase	136.90	0.00013327039960981	0.00291328984962392
19	PC(20:5(5Z,8Z,11Z,14Z,17Z)/15:0)	LSYAJRTVZQKSRW-BPFCGSCHSA-N	2.26	766.53	C_43_H_76_NO_8_P	Level2	[M + H]^+^	NGM_pbdatabase	37.30	0.000131349853019085	0.00288377146395841
20	5-Aminolevulinic acid	ZGXJTSGNIOSYLO-UHFFFAOYSA-N	3.92	130.05	C_5_H_9_NO_3_	Level1	[M − H]^−^	NGM_indatabase	206.80	0.000130705114434955	0.00287378737242506
21	4-Hydroxyproline	PMMYEEVYMWASQN-DMTCNVIQSA-N	3.89	130.05	C_5_H_9_NO_3_	Level1	[M − H]^−^	NGM_indatabase	206.80	0.000130705114434955	0.00287378737242506
22	LPE(P-20:0)	GBHFCJJMZAWCCH-HFQDTZRISA-N	2.72	494.36	C_25_H_52_NO_6_P	Level2	[M + H]^+^	NGM_indatabase	108.80	0.000124537269727203	0.00277060978630726
23	2-Acetylthiazole	MOMFXATYAINJML-UHFFFAOYSA-N	2.20	128.01	C_5_H_5_NOS	Level2	[M + H]^+^	NGM_pbdatabase	176.10	0.000122639648778696	0.00274548001088054
24	13(S)-HODE	HNICUWMFWZBIFP-IRQZEAMPSA-N	3.93	295.22	C_18_H_32_O_3_	Level1	[M − H]^−^	NGM_indatabase	25.10	0.000119771206449244	0.00270478532331431
25	Syrosingopine	ZCDNRPPFBQDQHR-MESICKPHSA-N	2.30	667.28	C_35_H_42_N_2_O_11_	Level2	[M + H]^+^	NGM_pbdatabase	151.90	0.000118284980768663	0.00268191542865314
26	Leu-Glu	NFNVDJGXRFEYTK-SFYZADRCSA-N	2.28	261.14	C_11_H_20_N_2_O_5_	Level2	[M + H]^+^	NGM_pbdatabase	240.20	0.000115954596740908	0.00265243798473811
27	Perfluorobutanoic acid	YPJUNDFVDDCYIH-UHFFFAOYSA-N	2.63	212.98	C_4_HF_7_O_2_	Level2	[M − H]^−^	NGM_pbdatabase	48.10	0.000115604273492558	0.00264656218353738
28	Isopongaflavone	DPAGRPSAFDXQDN-UHFFFAOYSA-N	2.74	335.12	C_21_H_18_O_4_	Level2	[M + H]^+^	NGM_pbdatabase	158.40	0.000108712451782357	0.0025422195887899
29	Ile-Leu	JWBXCSQZLLIOCI-GUBZILKMSA-N	3.97	245.18	C_12_H_24_N_2_O_3_	Level1	[M + H]^+^	NGM_indatabase	98.20	0.000107460359396008	0.00252335814074259
30	Leu-Ile	AZLASBBHHSLQDB-GUBZILKMSA-N	3.97	245.18	C_12_H_24_N_2_O_3_	Level1	[M + H]^+^	NGM_indatabase	98.20	0.000107460359396008	0.00252335814074259
31	4-Amino-6-methoxy-3-quinolinecarboxylic acid	GCQXPAGPGGXAAD-UHFFFAOYSA-N	2.74	219.07	C_11_H_10_N_2_O_3_	Level2	[M + H]^+^	NGM_pbdatabase	30.60	0.000106601514065492	0.00251780506824076
32	Lauroylcarnitine	FUJLYHJROOYKRA-QGZVFWFLSA-N	3.89	344.27	C_19_H_37_NO_4_	Level1	[M + H]^+^	NGM_indatabase	109.40	0.000105891164668112	0.00250939907300106
33	12′-Apozeaxanthin	PAUIQDPAEDELMC-HEZGKBSMSA-N	2.10	366.26	C_25_H_34_O_2_	Level2	[M] + *	NGM_pbdatabase	108.20	0.000105790955529732	0.00250912401142921
34	4-Acetamidobutyric acid	UZTFMUBKZQVKLK-UHFFFAOYSA-N	3.65	144.06	C_6_H_11_NO_3_	Level1	[M − H]^−^	NGM_indatabase	120.30	0.000103687230268348	0.00247581675714111

### 3.4 WLWDS-11 alters metabolic pathways underlying serum metabolite profiles in MIRI mice

**Figure 4:**
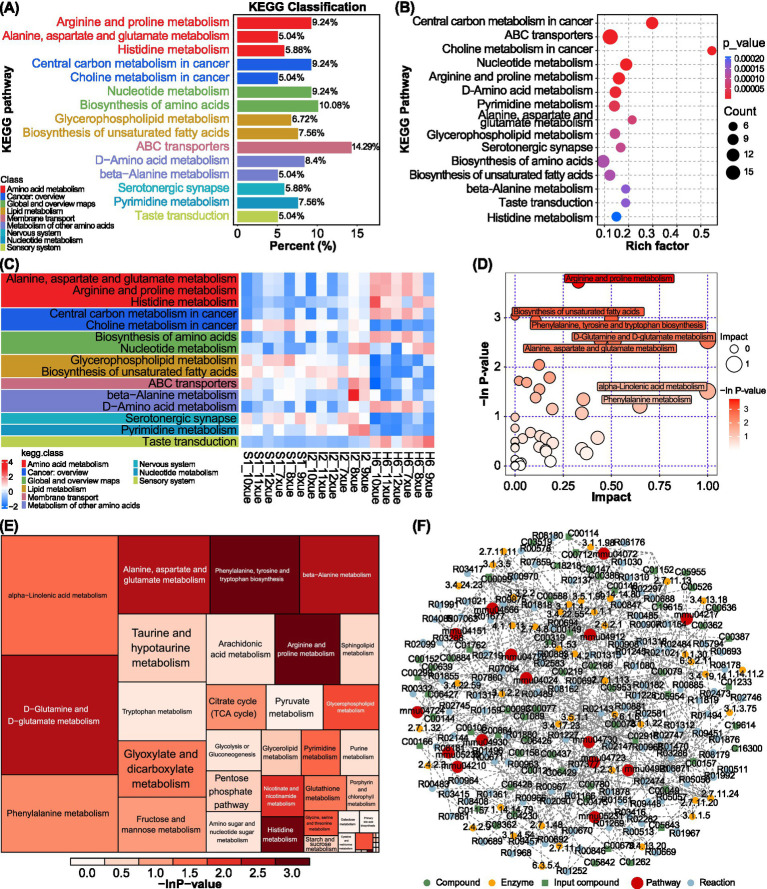
KEGG pathway enrichment analysis reveals WLWDS-11-mediated regulation of metabolic and signaling pathways in MIRI mice. **(A,B)** KEGG enrichment map of the differential metabolites in each group. **(C)** KEGG heat map of differential metabolites in each group. **(D,E)** Pathway analysis charts for each group. **(F)** Regulatory network analysis diagram of each group. (S is the Sham group, I is the MIRI group, and H is the WLWDS-11 high-dose group).

We will further explore the protective mechanism of WLWDS-11 regulating MIRI mice, and then analyze the related metabolic pathways. KEGG pathway analysis revealed that differential metabolites (DMs) were mainly involved in key metabolic pathways such as cofactor biosynthesis, ABC transporters, and amino acid biosynthesis (Figures 4A,B). The pathway-level heatmap further illustrated that MIRI induction disturbed endogenous metabolite patterns, an effect that was partially ameliorated by WLWDS-11-H treatment, normalizing the profile toward the sham group state (Figure 4C). Further screening beyond initial KEGG annotation revealed several core pathways most associated with the metabolic differences, including arginine and proline metabolism and the biosynthesis of unsaturated fatty acids (Figures 4D,E). The functional interplay within this metabolic network was visualized in a comprehensive diagram (Figure 4F), which maps reactions, enzymes, and metabolites to illustrate how different conditions target specific nodes, thereby causing perturbations that propagate across interconnected pathways.

### 3.5 WLWDS-11 rescues pathological alterations in the fecal microbiota of MIRI mice

**Figure 5:**
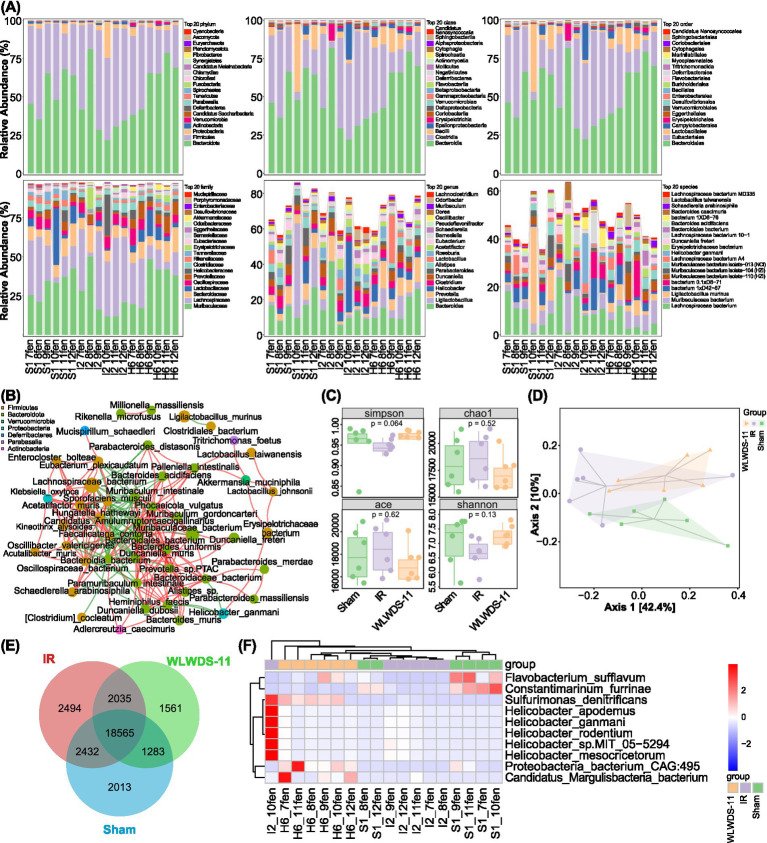
WLWDS-11 modulates gut microbiota composition, diversity, and key species abundance in MIRI mice. **(A)** Histogram of species classification at the phylum, class, order, family, genus, and species levels of each group. **(B)** Association network diagram of dominant microorganisms at the species level. **(C)** Result of species alpha diversity analysis. **(D)** Two-dimensional sequencing plot of the sample for the PCoA analysis of the species. **(E)** Species Venn diagram. **(F)** Species difference analysis. (S is the Sham group, I is the MIRI group, and H is the WLWDS-11 high-dose group).

To evaluate the effect of WLWDS-11 on the fecal microbiota, we profiled its composition and abundance from phylum to species level. Changes in the microbial community structure across experimental groups are characterized by composition bar charts of the top 20 dominant species at each taxonomic level (Figure 5A). At the phylum level, the sham group was dominated by *Bacteroidota*, *Bacillota*, *Pseudomonadota*, and *Campylobacterota*. In comparison, MIRI model mice showed a significant decrease in *Bacteroidota* and *Bacillota* (specifically, Bacilli), while *Pseudomonadota* and *Campylobacterota* increased significantly. In the WLWDS-11-H treatment group, *Bacillota*, *Pseudomonadota*, and *Campylobacterota* showed a decreasing trend, while *Bacteroidota* increased significantly and approached the levels in sham mice. At the class level, *Clostridia* and *Erysipetotrichia* were significantly increased in MIRI model versus sham mice; following WLWDS-11-H treatment, both classes were observed to decrease significantly, while *Bacteroidia* and *Bacilli* exhibited a significant increase.

Order-level analysis revealed that *Eubacteriales* and *Lactobacillales* predominated in the sham group; *Lactobacillales* decreased significantly in MIRI mice, with *Eubacteriales* showing an increasing trend. The WLWDS-11-H treatment improved the above situation, leading to a significant increase in *Lactobacillales* and a decreasing trend in *Eubacteriales*. Meanwhile, family-level analysis identified *Muribaculaceae*, *Lachnospiraceae*, and *Bacteroidaceae* as the dominant bacteria in sham mice, whereas MIRI model establishment produced a significant reduction in *Muribaculaceae* and *Lactobacillaceae*, but an increase in *Lachnospiraceae* and *Bacteroidaceae*. Relative to MIRI mice, treatment with WLWDS-11-H led to a significant increase in *Muribaculaceae* and a decreasing trend for *Lachnospiraceae*.

In terms of genera, *Bacteroides*, *Helicobacter*, and *Clostridium* were dominant in the sham group. Following model establishment, MIRI mice showed a decreasing trend for *Bacteroides*, but *Helicobacter* and *Clostridium* increased significantly. The above situation was improved in WLWDS-11-H mice, with an upward trend for *Bacteroides*, while *Helicobacter* and *Clostridium* decreased significantly.

Species-level results identified *Lachnospiraceae bacterium*, *Muribaculaceae bacterium*, and *Bacterium 1×D42-87* as predominant in the sham group. In comparison, *Lachnospiraceae bacterium* and *Bacterium 1×D42-87* were significantly higher in MIRI model mice, whereas *Muribaculaceae bacterium* showed a decreasing trend. After WLWDS-11-H treatment, these changes were attenuated: *Lachnospiraceae bacterium* and *Bacterium 1×D42-87* decreased significantly, while *Muribaculaceae bacterium* showed an increasing trend. As shown in Figure 5B, at the phylum level, Firmicutes (orange) and Bacteroidota (olive green) display many nodes and dense connections, defining them as “dominant gates” of the community that may play a core role in maintaining the structure and function of the microbiota. The connections between species of various phyla, such as Verrucomicrobia and Bacillota, and Bacteroidota and Pseudomonadota, reflect trans-phylum level interactions that may influence community stability. For instance, the green connection (positive correlation) between *Akkermansia muciniphila* (mint green Verrucomicrobia) and *Bacteroides acidifaciens* (olive green, Bacteroidota) suggests a degree of synergy between these two species in their metabolic pathways. WLWDS-11-H treatment counteracted the MIRI-induced reduction in microbial abundance and diversity, as evidenced by the species accumulation and rank-abundance curves (Figure 5C). The plateauing curves confirm that sampling was adequate and community richness was high. Otherwise, PCoA plot based on Bray–Curtis distance revealed that WLWDS-11-H treatment restored the fecal microbiota structure perturbed by MIRI, making it closely resemble that of the sham group (Figure 5D). This clear separation in beta diversity underscores the treatment’s efficacy in normalizing the overall microbial community composition. A Venn diagram showed 18,565 species were shared across all groups (Figure 5E). Abundance composition was normalized at each taxonomic level for each sample, then abundance differences of each taxonomic unit across sample groups were compared to evaluate whether the differences were statistically significant (see Table 3). To identify group-specific taxa, we performed differential abundance analysis using DESeq2 and confirmed the results with LEfSe and ANCOM2. Notably, several rare taxa (e.g., Chloracidobacterium) were enriched in MIRI mice and validated against the GTDB database, potentially indicating intestinal dysbiosis. The abundance patterns of the top 50 significantly different taxa are displayed in a heatmap (Figure 5F).

**Table 3:** Table of species grace differences.

Taxon	Groups	logFC	SE	adj. *p*-values
k__Bacteria;p__Actinobacteria;c__Actinomycetia;o__Micrococcales;f__Microbacteriaceae;g__Amnibacterium	Sham vs. IR	3.646	0.853	0.026
k__Bacteria;p__Bacteroidota;c__Bacteroidia;o__Bacteroidales;f__Muribaculaceae;g__Heminiphilus	Sham vs. IR	3.343	0.8399	0.04675
k__Bacteria;p__Acidobacteria;c__Blastocatellia;o__unclassified;f__unclassified;g__Chloracidobacterium	Sham vs. WLWDS-11	1.97	0.4893	0.03642
k__Bacteria;p__Proteobacteria;c__Gammaproteobacteria;o__Enterobacterales;f__Enterobacteriaceae;g__Kosakonia	Sham vs. WLWDS-11	−1.669	0.4398	0.04747
k__Bacteria;p__Bacteroidota;c__Flavobacteriia;o__Flavobacteriales;f__Flavobacteriaceae;g__Constantimarinum	Sham vs. WLWDS-11	−2.354	0.547	0.02164
k__Eukaryota;p__Zoopagomycota;c__Zoopagomycetes;o__Zoopagales;f__Piptocephalidaceae;g__Syncephalis	Sham vs. WLWDS-11	−2.383	0.6171	0.04747
k__Bacteria;p__Proteobacteria;c__Epsilonproteobacteria;o__Campylobacterales;f__Helicobacteraceae;g__Helicobacter;s__Helicobacter_ganmani	Sham vs. WLWDS-11	7.369	0.6652	0
k__Bacteria;p__Proteobacteria;c__Epsilonproteobacteria;o__Campylobacterales;f__Helicobacteraceae;g__Helicobacter;s__Helicobacter_rodentium	Sham vs. WLWDS-11	5.052	0.6987	8.651 × 10^−10^
k__Bacteria;p__Proteobacteria;c__Epsilonproteobacteria;o__Campylobacterales;f__Thiovulaceae;g__Sulfurimonas;s__Sulfurimonas_denitrificans	Sham vs. WLWDS-11	4.901	0.9732	0.0003423
k__Bacteria;p__Candidatus_Margulisbacteria;c__unclassified;o__unclassified;f__unclassified;g__unclassified;s__Candidatus_Margulisbacteria_bacterium	Sham vs. WLWDS-11	4.021	0.8906	0.003782
k__Bacteria;p__Proteobacteria;c__Epsilonproteobacteria;o__Campylobacterales;f__Helicobacteraceae;g__Helicobacter;s__Helicobacter_apodemus	Sham vs. WLWDS-11	3.97	0.6855	8.388 × 10^−6^
k__Bacteria;p__Proteobacteria;c__Epsilonproteobacteria;o__Campylobacterales;f__Helicobacteraceae;g__Helicobacter;s__Helicobacter_mesocricetorum	Sham vs. WLWDS-11	3.824	0.7252	0.0001202
k__Bacteria;p__Proteobacteria;c__Epsilonproteobacteria;o__Campylobacterales;f__Helicobacteraceae;g__Helicobacter;s__Helicobacter_sp.MIT_05–5,294	Sham vs. WLWDS-11	2.69	0.6286	0.00963
k__Bacteria;p__Bacteroidota;c__Flavobacteriia;o__Flavobacteriales;f__Flavobacteriaceae;g__Constantimarinum;s__Constantimarinum_furrinae	Sham vs. WLWDS-11		0.6244	0.04962
k__Bacteria;p__Bacteroidota;c__Flavobacteriia;o__Flavobacteriales;f__Flavobacteriaceae;g__Flavobacterium;s__Flavobacterium_sufflavum	Sham vs. IR	4.474	0.9133	0.001545
k__Bacteria;p__Proteobacteria;c__Epsilonproteobacteria;o__Campylobacterales;f__Helicobacteraceae;g__Helicobacter;s__Helicobacter_rodentium	Sham vs. IR	−6.299	0.9663	2.276 × 10^−7^
k__Bacteria;p__Proteobacteria;c__unclassified;o__unclassified;f__unclassified;g__unclassified;s__Proteobacteria_bacterium_CAG:495	WLWDS-11vs. IR	−3.857	0.7343	0.0005783

### 3.6 WLWDS-11 treatment-induced changes in gut microbiota in MIRI mice

**Figure 6:**
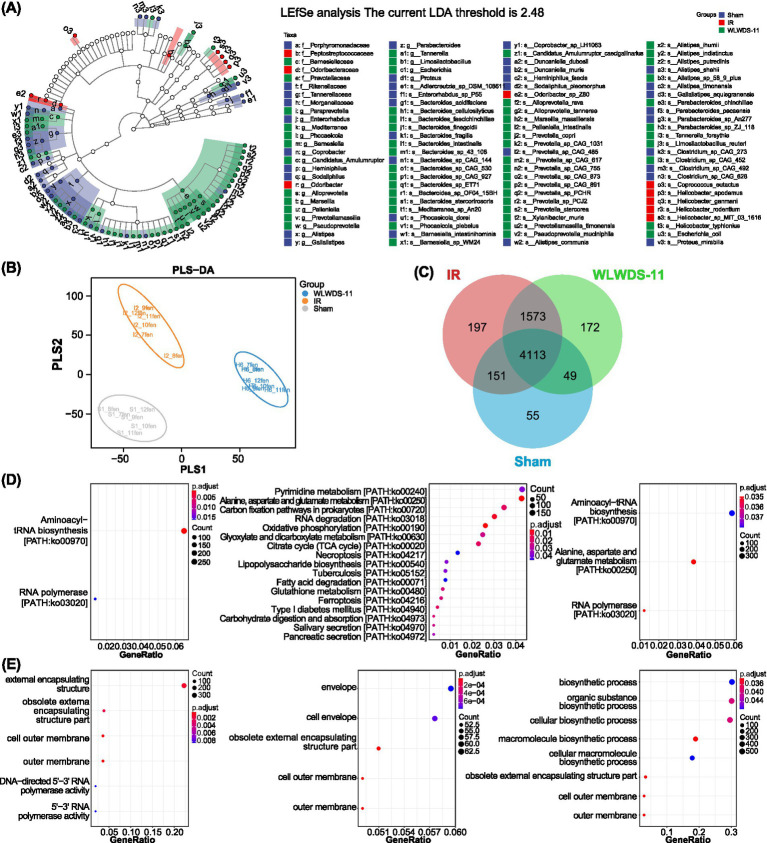
LEfSe-based identification of differential gut microbial taxa and functional enrichment analysis (KEGG/GO) in MIRI mice following WLWDS-11 intervention. **(A)** Branch of LEfSe species. **(B)** Ranking diagram of PLS-DA samples of species composition. **(C)** Venn diagram of functional taxa. **(D)** Dot plot of KEGG enrichment analysis, from left to right, Sham group and MIRI group, MIRI group and WLWDS-11 group, Sham group and WLWDS-11 group. **(E)** Dot plot of GO enrichment analysis, from left to right, the Sham group is compared with the MIRI group, the MIRI group is compared with the WLWDS-11 group, and the Sham group is compared with the WLWDS-11 group.

Both LEfSe and PLS-DA analyses indicated that WLWDS-11 treatment restored a gut microbiota composition resembling the sham group, in contrast to the distinct profile of MIRI model mice. LEfSe identified *Flavobacterium* and *Constantimarinum* as key taxa associated with the sham group (Figure 6A), while PLS-DA confirmed clear separations among all groups, supporting the robustness of the classification model (Figure 6B). Moreover, Microbial functional analysis demonstrated that WLWDS-11-H treatment shifted the functional profile of MIRI mice toward that of the sham group. While the sham and MIRI model groups shared enrichment in pathways like aminoacyl-tRNA biosynthesis, the MIRI model vs. WLWDS-11-H comparison revealed unique alterations in energy and amino acid metabolism (e.g., oxidative phosphorylation, alanine/aspartate/glutamate metabolism). Similarly, GO analysis showed that WLWDS-11-H treatment was associated with a recovery of biosynthetic processes, in contrast to the outer membrane-related terms enriched in MIRI mice (Figures 6C–E).

### 3.7 Multiomics integration analysis study on the regulation of flora-metabolite interaction in MIRI by WLWDS-11

**Figure 7:**
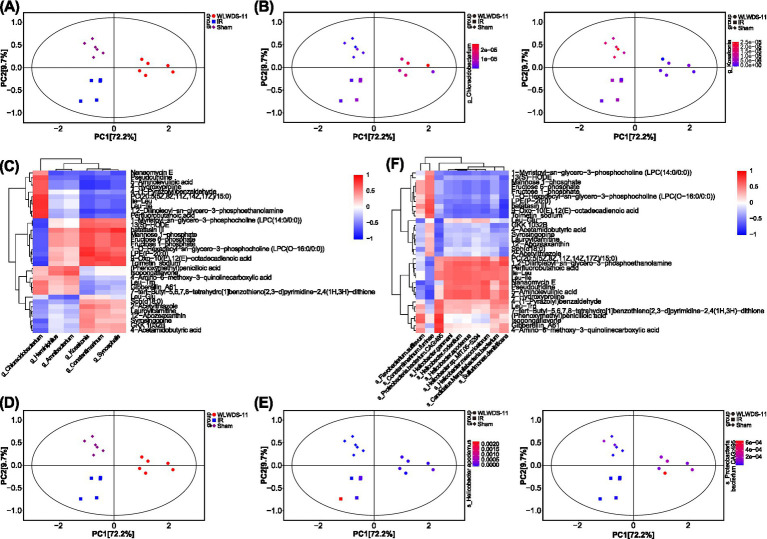
Integrated IPCA/PCA visualization and correlation analysis of gut microbiota-metabolite interactions in MIRI mice regulated by WLWDS-11. **(A)** Dispersion point plot of PCA associated with metabolites at the genus level. IPCA diagram of metabolism in each group at the level of genus **(B,C)** Genus level for correlation analysis clustering. **(D)** Dispersion point diagram of PCA associated with metabolites at the species level. IPCA diagram of metabolic metabolism in each group at the level of species **(E,F)** Species-level clustering heat map for correlation analysis.

By elucidating the relationships between metabolites, microorganisms, and cardiac pathology markers, this integrated approach holds promise not only for identifying novel clinical biomarkers but also for advancing our understanding of the underlying disease mechanisms. Correlation analysis was conducted via PCA, to discern both the clustering degree of samples within groups and the broader distribution trends for samples across groups. By comparing the two omics-derived maps, differences can be seen in the trends for microbiota community structure and metabolite profile, as illustrated in Figures 7A,D. In these graphics, interactive PCA (IPCA) visualization is employed to show clustering within and between groups of a single omics dataset, as well as the overall distribution trend of samples within and between the two omics profiles. The color depth of sample points reflects the magnitude of indicator values, showing the trend of this variable in the sample group. As shown in Figures 7B,E, the closer the distance between samples in the sham and WLWDS-11-H groups, the more similar the measured profiles of microbiota and metabolites; the results indicate that the genera *Chloracidobacterium* and *Kosakonia* were present at similar levels both experimental groups. Conversely, the greater the distance between sham, MIRI model, and WLWDS-11-H samples, the greater the differences between microbiota and metabolite profiles. In the MIRI model group (squares), red symbols are present, denoting samples with the highest abundance, while in the WLWDS-11-H (circles) and sham (diamonds) groups, most samples are blue/purple, indicating low abundance. Given the high explanatory power of PC1, it seems plausible to speculate that the difference in abundance of *Helicobacter apodemus* is the main reason for separation of the MIRI model mice from the WLWDS-11-H and sham groups (i.e., because this species is enriched in the model group and scarce in the others). It is worth noting that significant correlations between differential microbes and metabolites were identified via Spearman analysis and hierarchical clustering (Figures 7C,F). Key genus-level associations linked Kosakonia, Constantimarinum, and Syncephalis to phospholipids and sugar phosphates. At the species level, a cluster of Helicobacter species, along with Flavobacterium sufflavum and Candidatus Margulisbacteria bacterium, showed strong correlations with specific glycerophospholipids, dipeptides, and other metabolites.

### 3.8 WLWDS-11 inhibits Prevotella enrichment to ameliorate lipid metabolism dysregulation in MIRI

**Figure 8:**
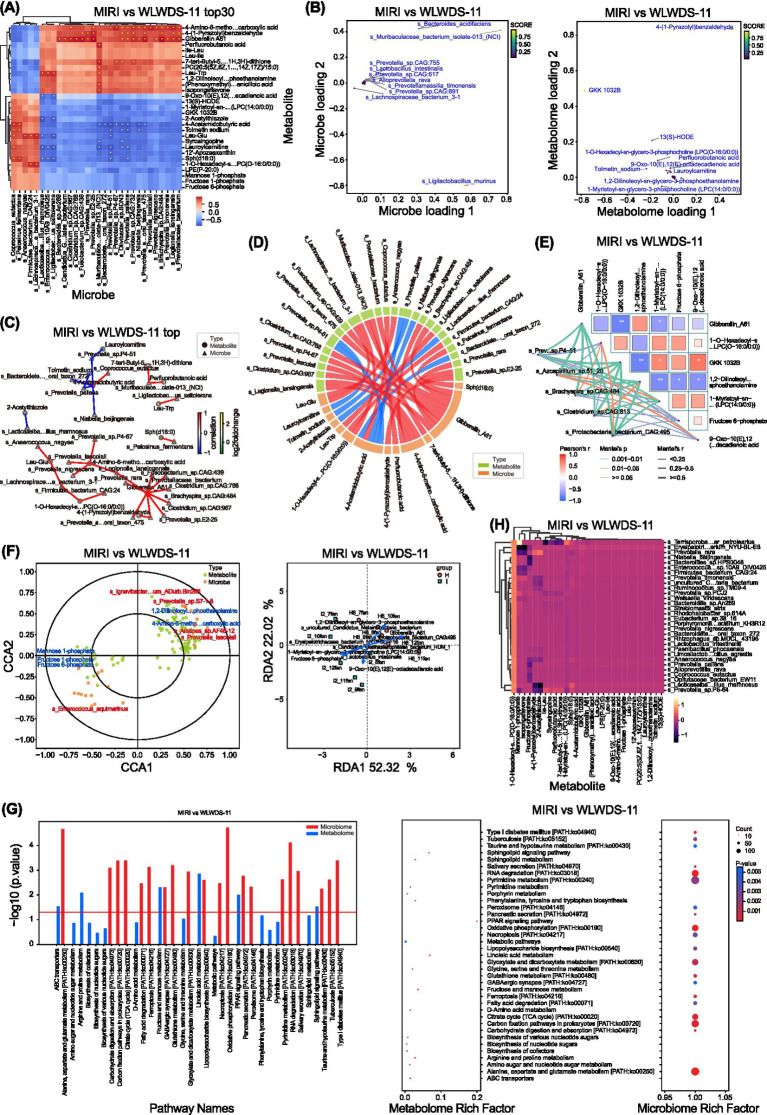
Key regulators, microbiota-metabolite crosstalk, and functional pathway enrichment in the gut microbiota-metabolite axis of MIRI mice treated with WLWDS-11. **(A)** Clustering heatmap of the top 30 microbes and metabolites between MIRI and WLWDS-11 groups. **(B)** O2PLS loadings plot of microbes and metabolites discriminating MIRI and WLWDS-11 groups. **(C)** Microbe-metabolite correlation network diagram. **(D)** Chord diagram of microbe-metabolite correlations. **(E)** Mantel test heatmap. **(F)** Ordination plot from CCA analysis and RDA analysis. **(G)** List of significantly enriched KEGG pathways. Bubble chart of KEGG pathway enrichment. **(H)** Integrated heatmap of microbes and metabolites in MIRI and WLWDS-11 groups.

To assess the influence of MIRI and WLWDS-11 treatment on the gut microbiota, we conducted metagenomic sequencing of fecal samples. Cluster analysis based on the top 30 most differentially abundant species clearly separated the MIRI and WLWDS-11 groups (Figure 8A), indicating a substantial shift in microbial structure following treatment. Notably, several species from the genus Prevotella formed a distinct enrichment cluster in the MIRI group. Concordant with these microbial alterations, metabolomic profiles also clearly distinguished the two groups. The MIRI group exhibited characteristic accumulation of diverse lipid molecules, including oxidized lipids, lysophospholipids, and intermediates of fatty acid β-oxidation.

To identify the key species responsible for this separation within the statistical model, we performed O2PLS analysis. The results highlighted several Prevotella species—most prominentlys Prevotella_sp.CAG:617—as the primary drivers differentiating the MIRI from the WLWDS-11 group (Figure 8B), establishing their pivotal role in the underlying mechanism. Metabolites such as Lauroylcarnitine and 13(S)-HODE contributed most strongly to group separation in the model, suggesting that disrupted energy metabolism and oxidative stress represent core metabolic characteristics of the MIRI condition.

We further explored functional relationships between the microbiota and host metabolism by constructing correlation networks. Figure 8C visually represents significantly correlated microbe–metabolite pairs, revealing close associations between Lauroylcarnitine and several Prevotell species. The chord diagram in Figure 8D further delineated these complex interactions, emphasizing strong connections between the Prevotella genus and key metabolite modules. The Mantel test provided statistical support for a global association, confirming a significant correlation between gut microbiota composition and serum metabolomic profiles (Figure 8E), indicating their coordinated variation.

To understand the biological implications of these changes, we performed KEGG pathway enrichment analysis. The most significantly enriched pathways included necroptosis, ferroptosis, and oxidative phosphorylation (Figure 8G). The bubble chart illustrates that these pathways are both statistically significant and supported by high gene coverage. To verify that the MIRI/WLWDS-11 grouping was the principal factor driving the observed multi-omics variations, we employed constrained ordination analysis. Figures 8F collectively confirm that experimental grouping constitutes the major environmental factor explaining variance in both microbial and metabolomic data.

Finally, we constructed an integrated heatmap to offer a comprehensive overview of the dataset. Figure 8H simultaneously displays microbial and metabolomic profiles across all samples, clearly revealing a co-occurring “pathogenic module” in the MIRI group. This module—comprising Prevotella, oxidized lipids, and markers of energy metabolism dysfunction—provides compelling visual support for our hypothesis.

### 3.9 WLWDS-11 regulates the oxidative phosphorylation and necroptosis-related indicators in MIRI mice

**Figure 9:**
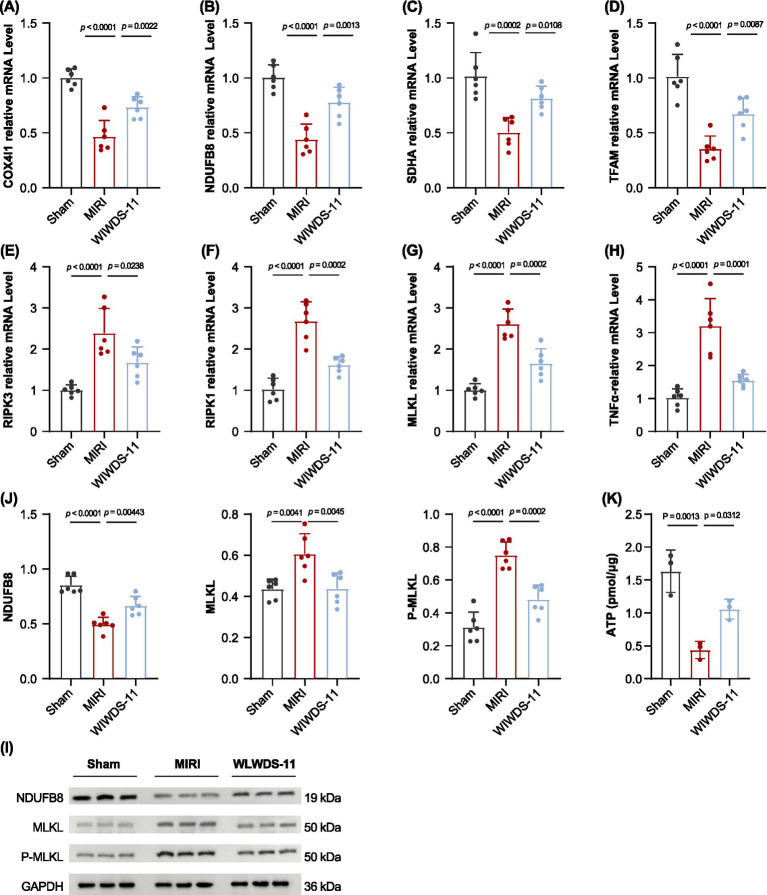
WLWDS-11 regulates mitochondrial function-related genes, necroptosis pathway components, and inflammatory factor expression at mRNA, protein levels, ATP levels in MIRI mice. **(A)** COX4 mRNA levels. **(B)** NDUFB8 mRNA levels. **(C)** SDHA mRNA levels. **(D)** TFAM mRNA levels. **(E)** MLKL mRNA levels. **(F)** RIPK1 mRNA levels. **(G)** RIPK3 mRNA levels. **(H)** TNF-α mRNA levels. Gene expression was normalized to β-actin mRNA levels (*n* = 6 per group). **(I,J)** The effects of WLWDS-11 on the expression of NDUFB8, MLKL, and P-MLKL in liver tissues of MIRI mice were detected by western blot analysis. **(K)** ATP levels in myocardial tissue across three groups (*n* = 3 per group).

To investigate the effects of MIRI and WLWDS-11 intervention on mitochondrial function and necroptosis in myocardial tissue, we detected the mRNA levels of mitochondrial function-related genes (COX4I1, NDUFB8, SDHA, TFAM) and necroptosis-related genes (RIPK1, RIPK3, MLKL, TNF-α) by qRT-PCR, and verified the protein expression of NDUFB8, MLKL, and phosphorylated MLKL (P-MLKL) by WB. β-actin was used as the internal reference gene for qRT-PCR, and GAPDH (36 kDa) was used as the loading control for WB. Statistical comparisons were performed between groups, with significant differences marked as indicated (Figures 9A–H for qPCR, Figures 9I,J for WB).

Mitochondrial dysfunction is a key pathological feature of MIRI, and the expression of mitochondrial function-related genes directly reflects the integrity and functional status of the mitochondrial respiratory chain. As shown in qRT-PCR results (Figures 9A–D), compared with the Sham group, the relative mRNA levels of COX4I1, NDUFB8, SDHA and TFAM in the MIRI group were significantly downregulated (all *p* < 0.0001 for Sham vs. MIRI). Notably, WLWDS-11 intervention significantly reversed these downregulatory trends: COX4I1, NDUFB8, SDHA, and TFAM mRNA levels were all markedly elevated compared with the MIRI group, restoring to near the baseline levels of the Sham group. These results indicate that MIRI severely impairs mitochondrial respiratory chain function and mtDNA regulatory capacity, while WLWDS-11 can effectively improve mitochondrial function by upregulating the transcription of these key genes.

Necroptosis, mediated by RIPK1/RIPK3/MLKL signaling pathway, plays a crucial role in the pathological process of MIRI. Our qRT-PCR results (Figures 9E–H) showed that compared with the Sham group, the relative mRNA levels of RIPK1, RIPK3, MLKL, and the pro-inflammatory cytokine TNF-α in the MIRI group were dramatically upregulated (all *p* < 0.0001 for Sham vs. MIRI), suggesting that MIRI triggers intense necroptosis and inflammatory response in myocardial tissue. However, WLWDS-11 intervention significantly suppressed the overexpression of these necroptosis-related genes: RIPK1, RIPK3, MLKL and TNF-α mRNA levels were significantly reduced compared with the MIRI group, indicating that WLWDS-11 can alleviate MIRI-induced myocardial necroptosis and inflammation by inhibiting the activation of the RIPK1/RIPK3/MLKL pathway.

To further confirm the regulatory effect of WLWDS-11 at the protein level, WB analysis was performed (Figures 9I,J). The WB bands were grouped as Sham, MIRI, and WLWDS-11, with GAPDH (36 kDa) as the internal reference. The results showed that the protein expression level of NDUFB8 (19 kDa) in the MIRI group was significantly lower than that in the Sham group (*p* < 0.0001), while WLWDS-11 intervention significantly increased NDUFB8 protein expression, which was consistent with the qRT-PCR results. For necroptosis-related proteins, the protein levels of MLKL (50 kDa) and P-MLKL (50 kDa) in the MIRI group were significantly higher than those in the Sham group. In contrast, WLWDS-11 intervention significantly downregulated the protein expression of MLKL and P-MLKL compared with the MIRI group. These WB results further verified that WLWDS-11 can effectively improve mitochondrial function and inhibit necroptosis in MIRI-induced myocardial tissue.

To illustrate the changes in myocardial energy metabolism, Figure 9K presents the levels of ATP in myocardial tissue across three experimental groups. Results showed that the Sham group exhibited the highest myocardial ATP level, which reflects the normal energy metabolic status of intact cardiac tissue. In contrast, the MIRI group displayed a significant reduction in myocardial ATP level compared with the Sham group, indicating that ischemia-reperfusion injury impairs the energy-producing function of myocardial tissue, thereby leading to decreased intracellular ATP synthesis or increased ATP consumption. Notably, relative to the MIRI group, the WLWDS-11 group showed a marked recovery of myocardial ATP level, suggesting that WLWDS-11 can alleviate MIRI-mediated impairment of myocardial energy metabolism and thus promote the restoration of ATP levels.

Collectively, these findings demonstrate that MIRI induces myocardial damage through dual mechanisms: impairing mitochondrial function by downregulating mitochondrial respiratory chain and regulatory genes, and activating necroptosis via upregulating the RIPK1/RIPK3/MLKL signaling pathway and pro-inflammatory cytokine TNF-α. WLWDS-11 exerts a protective effect against MIRI by reversing the abnormal expression of these key genes and proteins, which provides strong molecular evidence for its potential as a therapeutic agent for MIRI.

## 4 Discussion

Current research on myocardial ischemia-reperfusion injury (MIRI) recognizes it as a multifaceted process involving an intricate network of pathways. Key mechanisms include inflammation initiated by DAMPs, mitochondrial dysfunction—marked by energy failure and mPTP opening—and regulated cell death such as necroptosis and ferroptosis. Although interventions like ischemic conditioning have demonstrated strong preclinical potential, their clinical translation remains challenging. However, studies have allowed researchers to discover bidirectional information exchange and regulatory networks between resident microorganisms, the gut, and the heart, known as the “microbiota-gut-heart axis” (MGHA) (Kamo et al., 2017). Intestinal endothelial dysfunction and metabolic imbalance may lead to cardiac dysfunction, inflammation, malnutrition, and other diseases, while an imbalance in the gut microbiota promoted the growth of bacteria that produce toxic metabolites. Prior studies have shown that the gut microbiota significantly influences the pathophysiology of CVD, such as coronary heart disease and MIRI (Carnevale et al., 2020) and that gut metabolites may even trigger or exacerbate MIRI (Dong et al., 2025). Therefore, in our study, we found that WLWDS-11 has a positive effect on the intestinal microbial environment, promoting the real ecology, weakening the MIRI pathology, and finally protecting the heart by inhibiting the proliferation of pathogenic bacteria and promoting the growth of beneficial strains. More importantly, WLWDS-11 ameliorated MIRI-induced mitochondrial dysfunction by upregulating the expressions of COX4I1, NDUFB8, SDHA, and TFAM, and conferred cytoprotection by suppressing the RIPK1/RIPK3/MLKL pathway and TNF-α, thereby inhibiting necroptosis and the inflammatory response.

WLWDS-11 is a classic traditional Mongolian medicine formulation with a long history of clinical application in Inner Mongolia, China. Composed of multiple herbal ingredients, it is traditionally documented to exert pharmacological effects including warming the stomach, separating clear from turbid substances, nourishing the heart, and promoting blood circulation. WLWDS-11 is widely used for the treatment of CVDs including coronary heart disease, hypertension, and heart failure (Surongzabu, 2011). WLWDS-11 can suppress the expression of AT1R and promote the expression of AT2R in spontaneously hypertensive rats, thereby ameliorating hypertensive vascular remodeling (Genduxi et al., 2019). However, the potential role and underlying mechanism of WLWDS-11 in myocardial ischemia-reperfusion injury (MIRI) remain largely unelucidated. Therefore, in the present study, we further investigated the effects of WLWDS-11 on MIRI. In our study, compared to the sham surgery group, MIRI model mice displayed weight loss, significantly elevated heart rate, and ST-segment elevation. Histopathological analysis revealed early multifocal necrosis, which was replaced after 7 days by proliferating fibrous connective tissue and interstitial fibrosis. Extensive myocardial atrophy, decreased cell volume, widened intercellular spaces, and TUNEL-positive apoptosis with nuclear condensation and fragmentation were also observed, confirming successful model establishment. Pretreatment with WLWDS-11 for 4 weeks alleviated these pathological changes, with the most pronounced effects in the high-dose group (WLWDS-11-H). Therefore, we found that WLWDS-11 significantly protected the heart from MIRI-mediated heart damage. However, it is not clear whether WLWDS-11 can affect the function of intestinal flora, thus regulating MIRI. So, we studied this process further.

Among the trillions of commensal bacteria found in the human gastrointestinal tract, most are nonpathogenic and engage in mutual symbiosis with host cells (Luo, 2024). The gut microbiota participates in metabolism of food and helps maintain the intestinal barrier, prevent colonization by pathogens, and stimulate the development and maturation of mucosal immune structures (Knight and Girling, 2003; Rooks and Garrett, 2016). Most commensal species belong to the phyla Bacteroidota, Bacillota, Actinomycetota, and Pseudomonadota (Davenport et al., 2017). While the colon also harbors pathogenic *Escherichia coli*, *Salmonella* spp., and *Streptococcus* spp., among other pathogens (Hu and Zhang, 2020), commensal bacteria inhibit colonization by these species through competing for limited nutrients, thereby contributing to stability of the microbiota community structure. If changes in the host’s intestinal/extraintestinal environment lead to declining abundance of dominant microbial communities, the resultant dysbiosis will permit pathogenic bacteria to proliferate and cause disease. In this study, we found that WLWDS-11 administration reprogrammed the gut microbiota and shifted the metabolic landscape, as evidenced by distinct PCA clustering. Key metabolic alterations included disruptions in arginine/proline metabolism, unsaturated fatty acid biosynthesis, and aromatic amino acid pathways. Concurrently, the treatment favorably modulated the gut microbiota by enhancing Bacteroidota (e.g., Muribaculaceae) and Lactobacillales, while depleting potentially harmful taxa such as Campylobacterota, Bacillota (e.g., Lachnospiraceae), and the genera Helicobacter and Clostridium. Critically, alpha diversity analysis indicated that MIRI reduced both gut microbial abundance and diversity, which were restored by WLWDS-11 treatment. This shift was supported by beta diversity (PCoA), which showed a clear separation between groups and a reversal of the MIRI-induced microbiota composition toward the sham group profile. KEGG enrichment further implicated several cardiovascular-related pathways—such as aminoacyl-tRNA biosynthesis, oxidative phosphorylation, and alanine, aspartate, and glutamate metabolism—in the mechanism of WLWDS-11. Together, these findings suggest that WLWDS-11 alleviates MIRI by restoring gut microbiota composition and modulating key microbial metabolic and signaling pathways. Multi-omics integration is advantageous in studying microbiota–metabolite interactions, as exemplified through the present PCA and IPCA visualization results (Figures 7A,B,D,E), which indicated highly similar microbiota and metabolite patterns for the sham and WLWDS-11-H groups, in contrast to significant separation from the MIRI model group. This phenomenon hints at a close relationship between microbiota structural stability and metabolic homeostasis; for instance, the abundance of Chloracidobacterium and Kosakonia genera was similar in both groups, potentially participating in host physiological regulation by maintaining specific metabolic pathways (such as short-chain fatty acid synthesis). The distant distribution of the MIRI model group suggests that dysbiosis is accompanied by metabolic dysregulation, consistent with previous studies on the involvement of gut microbiota in metabolic diseases (Ma et al., 2023). Hierarchical clustering and correlation heat maps (Figures 7C,F) identified significantly associated bacterial communities and metabolites. At the genus level, strong correlations were observed between Kosakonia and Constantimarinum spp. and 1-myristoyl-sn-glycero-3-phosphocholine, as well as sugar phosphate derivatives (e.g., fructose-6-phosphate), suggesting a potential role for these bacteria in influencing physiological states by regulating host lipid metabolism and energy supply. At the species level, *H. ganmani* was associated with phosphatidylethanolamine (PE) phospholipids (e.g., 1,2-dilinoleoyl-sn-glycero-3-phosphoethanolamine), reflecting a potential regulatory role in the intestinal mucosal barrier.

Although this study has to some extent revealed an association between MIRI and gut microbiota, it also indicates that therapeutic formulations which promote beneficial microbiota structure can attenuate intestinal dysbiosis in MIRI mice. Nevertheless, further research is needed to elucidate the underlying mechanisms. The pathological mechanism of myocardial ischemia-reperfusion injury (MIRI) is complex and not yet fully elucidated, with mitochondrial dysfunction and abnormal activation of regulated cell death (RCD) recognized as core pathogenic links. Studies have shown that mitochondria are not only a key potential therapeutic target for MIRI, but mitochondrial homeostasis imbalance (including mitochondrial DNA damage, metabolic dysfunction, etc.) is also a vital driving force for the development of ischemia-reperfusion injury in cardiovascular diseases (Marin et al., 2020; Cheng et al., 2025; Zou et al., 2022; Liu et al., 2022). Meanwhile, the role and mechanism of regulated cell death in MIRI have become a research hotspot, and in-depth analysis of its molecular regulatory network can provide new insights for the prevention and treatment of MIRI. In addition, comprehensively clarifying the injury mechanisms of MIRI (such as oxidative stress, inflammatory response, mitochondrial dysfunction, etc.) is of great significance for optimizing clinical management strategies (He et al., 2022; Xiang et al., 2024).

To evaluate the effect of MIRI and WLWDS-11 treatment on intestinal flora, we sequenced the metagenome of fecal samples. Cluster analysis based on the top 30 most diverse species clearly separated MIRI group from WLWDS-11 group (Figure 8A), indicating that the microbial structure changed significantly after treatment. It is worth noting that several species of Prevost have formed unique enrichment clusters in MIRI group. Consistent with these microbial changes, the metabonomics spectrum also clearly distinguishes the two groups. MIRI group showed the characteristic accumulation of various lipid molecules, including oxidized lipids, lysophospholipids and fatty acid β-oxidation intermediates. In order to determine the key species causing this separation in the statistical model, we conducted O2PLS analysis.

## 5 Results

Several species of Prevost-most notably Prevotella_sp. CAG: 617-were highlighted as the main driving factors to distinguish MIRI group from WLWDS-11 group (Figure 8B), and their key roles in this mechanism were established. Metabolites such as Lauroylcarnitine and 13(S)-HODE have the strongest contribution to the separation between groups in the model, suggesting that energy metabolism disorder and oxidative stress represent the core metabolic characteristics of MIRI status. We further explored the functional relationship between flora and host metabolism by constructing correlation network. Figure 8C shows a significant correlation of microorganism-metabolite pairing visually, revealing the close relationship between Lauroylcarnitine and several Prevost’s bacteria. The chord diagram in Figure 8D further illustrates these complex interactions and emphasizes the strong connection between Prevost and key metabolite modules. Mantel test provided statistical support for the overall correlation, and confirmed that there was a significant correlation between intestinal flora composition and serum metabonomics spectrum (Figure 8E), indicating their coordinated changes. In order to understand the biological significance of these changes, we carried out KEGG pathway enrichment analysis. The most significantly enriched pathways include necrotic apoptosis, iron death and oxidative phosphorylation (Figure 8G). The bubble diagram shows that these pathways are statistically significant and supported by high gene coverage. In order to verify that MIRI/WLWDS-11 grouping is the main factor driving the observed multi-omics variation, we adopted constrained ranking analysis. Figure 8F confirms that experimental grouping is the main environmental factor to explain the variation of microbial and metabonomic data.

In order to study the effects of MIRI and WLWDS-11 intervention on mitochondrial function and necrotizing apoptosis in myocardial tissue, we detected the expression levels of mitochondrial function-related genes (COX4I 1, NDUFB 8, SDHA, TFAM) and necrotizing apoptosis-related genes (RIPK1, RIPK3, MLKL, TNF-α). Mitochondrial dysfunction is an important pathological feature of MIRI, and the expression of mitochondrial function-related genes directly reflects the integrity and functional status of mitochondrial respiratory chain. WLWDS-11 can effectively improve mitochondrial function by up-regulating the transcription of these key genes.

RIPK1/RIPK3/MLKL signaling pathway-mediated necroptosis is a key mechanism that exacerbates cardiomyocyte injury in myocardial ischemia-reperfusion injury (MIRI) and myocardial hypoxia/reoxygenation injury, and related regulatory strategies have become an important direction in MIRI protection research. Studies have confirmed that resveratrol can effectively inhibit necroptosis in myocardial hypoxia/reoxygenation injury and alleviate cardiomyocyte damage by mediating the regulation of the TNF-α/RIP1/RIPK3/MLKL pathway (Hu et al., 2021); while sevoflurane postconditioning can reduce MIRI-induced necroptosis and exert a myocardial protective effect by up-regulating O-GlcNAcyltransferase (OGT)-mediated O-GlcNAcylation of RIPK3 (Zhang et al., 2020). In addition, the ubiquitination of RIPK3 and MLKL, as core links in the regulation of necroptosis, the in-depth analysis of their molecular mechanisms provides a key theoretical basis for understanding how cells resist necroptosis-related damage, and also lays a foundation for the development of MIRI intervention strategies targeting this pathway (Arlowitz and van Wijk, 2021). The intervention of WLWDS-11 significantly inhibited the over-expression of these genes related to necrotic apoptosis. At the level of mRNA and protein, we further verified that WLWDS-11 can reduce MIRI-induced myocardial necrosis apoptosis and inflammation by inhibiting the activation of the RIPK 1/RIPK 3/MLKL pathway.

Collectively, our findings illustrate that MIRI induces myocardial damage through dual pathogenic mechanisms: it impairs mitochondrial function via downregulating the mitochondrial respiratory chain and its associated genes, while concurrently activating necroptosis through the RIPK1/RIPK3/MLKL pathway—triggered by the pro-inflammatory cytokine TNF-α. Crucially, the Mongolian medicine WLWDS-11 exerts cardioprotective effects against MIRI by reversing the aberrant expression of these key genes and proteins, thus providing robust molecular evidence for its clinical therapeutic potential. Future studies utilizing germ-free animal models, fecal microbiota transplantation (FMT), or metabolic flux analysis are required to validate the causal role of the gut microbiota in WLWDS-11-mediated cardioprotection.

## 6 Conclusion

This work has offered a new perspective on mechanistic studies of potential disease treatments through a multi-omics integration strategy. Future functional experiments could complement the present findings by further exploring the metabolic regulatory mechanisms of key microbial strains. In brief, in our research, we found for the first time that WLWDS-11 reverses MIRI-induced mitochondrial dysfunction by upregulating COX4I1, NDUFB8, SDHA, and TFAM. Its protective effects are also mediated by targeting the RIPK1/RIPK3/MLKL pathway and TNF-α, which concurrently inhibits necroptosis and inflammation.

## Data Availability

The data from this study are available in the NCBI and MetaboLights repositories, under accession numbers PRJNA1367916 and MTBLS13370, respectively.

## Glossary

MIRI: Myocardial ischemia-reperfusion injury
IHD: Ischemic heart disease
WLWDS-11: Wulanwendusu-11
CDDP: Danshen dripping pills
ECG: Electrocardiogram
CK: Creatine kinase
CK-MB: Creatine kinase-MB isoenzyme
LDH: Lactate dehydrogenase
cTnI: Cardiac troponin I
CTGF: Connective tissue growth factor
TGF-β1: Transforming growth factor beta-1‌
Gal-3: Galectin-3
TTC: Triphenyltetrazolium chloride
TdT: Terminal deoxynucleotidyl transferase
PCA: Principal component analysis
PCoA: Principal coordinate analysis
NMDS: Nonmetric multidimensional scaling
UPGMA: Unweighted pair group method with arithmetic mean
LEfSe: Linear discriminant analysis effect size
MGHA: Microbiota-gut-heart axis
GO: Gene Ontology
KEGG: Kyoto Encyclopedia of Genes and Genomes
