# Supplementary material for: Mongolian medicine Wulanwendusu-11 alleviates myocardial ischemia-reperfusion injury by modulating the intestinal microbiota and associated metabolic pathways
Source: Front Microbiol. 2026 Jan 9;16:1693472. doi: 10.3389/fmicb.2025.1693472 (PMC12828672; doi:10.3389/fmicb.2025.1693472)
Supplement: Supplementary file 1 [file Data_Sheet_1.DOCX]

**Supplementary Table 1. Characteristic Chemical Markers for WLWDS-11**

| Peak.No | Name | InChIKey | Formula | Class | rtmed | type |
| --- | --- | --- | --- | --- | --- | --- |
| 1 | Neocryptotanshinone | LGZFJHSOBYVDLA-JTQLQIEISA-N | C_19_H_22_O_4_ | Naphthalenes | 433.2 | POS |
| 2 | 1,6,6-trimethyl-8,9-dihydro-7H-naphtho[1,2-g]benzofuran-10,11-dione | HYXITZLLTYIPOF-UHFFFAOYSA-N | C_19_H_18_O_3_ | Naphthalenes | 487.4 | POS |
| 3 | Sodium 3-(3,4-dihydroxyphenyl)-2-hydroxypropanoate | PAFLSMZLRSPALU-MRVPVSSYSA-N | C_9_H_10_O_5_ | Phenylpropanoids | 405.2 | NEG |
| 4 | Quercetin | REFJWTPEDVJJIY-UHFFFAOYSA-N | C_15_H_10_O_7_ | Flavonoids | 345.2 | POS |
| 5 | Isorhamnetin | IZQSVPBOUDKVDZ-UHFFFAOYSA-N | C_16_H_12_O_7_ | Flavonoids | 364.4 | NEG |
| 6 | Kaempferol | IYRMWMYZSQPJKC-UHFFFAOYSA-N | C_15_H_10_O_6_ | Flavonoids | 369 | NEG |
| 7 | Prunin | DLIKSSGEMUFQOK-SFTVRKLSSA-N | C_21_H_22_O_10_ | Flavonoids | 314.8 | POS |
| 8 | 5-hydroxy-2-(4-hydroxyphenyl)-7-[(2S,3R,4S,5S,6R)-3,4,5-trihydroxy-6-(hydroxymethyl)tetrahydropyran-2-yl]oxy-chroman-4-one | DLIKSSGEMUFQOK-RGHIGTIISA-N | C_21_H_22_O_10_ | Flavonoids | 314.8 | POS |
| 9 | (2S)-5,7-dihydroxy-2-(4-hydroxyphenyl)-6-[(2S,3R,4R,5S,6R)-3,4,5-trihydroxy-6-(hydroxymethyl)tetrahydropyran-2-yl]chroman-4-one | QKPKGDDHOGIEOO-JVVVWQBKSA-N | C_21_H_22_O_10_ | Flavonoids | 239.8 | NEG |
| 10 | Dehydrocostus lactone | NETSQGRTUNRXEO-XUXIUFHCSA-N | C_15_H_18_O_2_ | Sesquiterpenoids | 440.7 | POS |
| 11 | Aurantio-obtusin β-D-glucoside | LQYQYAJWKXDTHR-PHVGODQESA-N | C_23_H_24_O_12_ | Polycyclic aromatic polyketides | 316 | POS |
| 12 | Myristic acid | TUNFSRHWOTWDNC-UHFFFAOYSA-N | C_14_H_28_O_2_ | Fatty Acids and Conjugates | 539.2 | NEG |
| 13 | Medicarpin | NSRJSISNDPOJOP-BBRMVZONSA-N | C_16_H_14_O_4_ | Isoflavonoids | 388.1 | POS |
| 14 | 1-(2,4-dihydroxyphenyl)-2-(4-methoxyphenyl)propan-1-one | CCOJFDRSZSSKOG-UHFFFAOYSA-N | C_16_H_16_O_4_ | Phenolic Ketones | 406.9 | NEG |
| 15 | Rosmarinic acid | DOUMFZQKYFQNTF-WUTVXBCWSA-N | C_18_H_16_O_8_ | Phenylpropanoids (C6-C3) | 304.5 | NEG |
| 16 | Procyanidin B2 | XFZJEEAOWLFHDH-NFJBMHMQSA-N | C_30_H_26_O_12_ | Flavonoids | 245.3 | POS |
| 17 | Procyanidin C1 | MOJZMWJRUKIQGL-XILRTYJMSA-N | C_45_H_38_O_18_ | Flavonoids | 254.6 | NEG |

**Supplementary Table 4. Key Quality Control Metrics of Metagenomic Sequencing Data**

| Sample ID | Reads Count | Bases Count(bp) | N(%) | GC(%) | Q20(%) | Q30(%) |
| --- | --- | --- | --- | --- | --- | --- |
| WLWDS-11-1 | 51620958 | 7683425348 | 0 | 44.04 | 99.54 | 97.63 |
| WLWDS-11-2 | 50639960 | 7533854952 | 0 | 44.68 | 99.49 | 97.46 |
| WLWDS-11-3 | 42747866 | 6365569788 | 0 | 43.77 | 99.51 | 97.5 |
| WLWDS-11-4 | 39239322 | 5850464605 | 0 | 45.18 | 99.52 | 97.53 |
| WLWDS-11-5 | 58065450 | 8643885396 | 0 | 43.84 | 99.53 | 97.62 |
| WLWDS-11-6 | 36978234 | 5505898226 | 0 | 43.78 | 99.48 | 97.41 |
| MIRI-1 | 54894528 | 8168937659 | 0 | 43.29 | 99.47 | 97.42 |
| MIRI-2 | 56881334 | 8514416589 | 0 | 46.62 | 99.57 | 97.63 |
| MIRI-3 | 55243778 | 8265798750 | 0 | 43.67 | 99.56 | 97.67 |
| MIRI-4 | 55243778 | 8265798750 | 0 | 43.67 | 99.56 | 97.67 |
| MIRI-5 | 40650110 | 6105949701 | 0 | 46.65 | 99.58 | 97.65 |
| MIRI-6 | 54117794 | 8107282823 | 0 | 47.53 | 99.57 | 97.61 |
| Sham-1 | 45492420 | 6775856255 | 0 | 46.74 | 99.54 | 97.53 |
| Sham-2 | 49828414 | 7452168681 | 0 | 46.03 | 99.56 | 97.64 |
| Sham-3 | 44353870 | 6659972213 | 0 | 46.93 | 99.61 | 97.83 |
| Sham-4 | 46208096 | 6914413305 | 0 | 44.94 | 99.58 | 97.72 |
| Sham-5 | 51964916 | 7727393019 | 0 | 44.08 | 99.51 | 97.53 |
| Sham-6 | 37762658 | 5286379621 | 0 | 44.8 | 99.46 | 97.21 |

**Supplementary Table 5. α-Diversity Indices of Gut Microbiota**

| Sample | simpson | chao1 | ace | shannon |
| --- | --- | --- | --- | --- |
| Sham-1 | 0.957351987004958 | 20067.5030959752 | 19680.0151835662 | 7.17763738488458 |
| Sham-2 | 0.972894157053039 | 21111.7408704352 | 20694.9658029332 | 7.5657120804459 |
| Sham-3 | 0.978321231718271 | 18960.4774358974 | 18823.1729905518 | 7.68143753296985 |
| Sham-4 | 0.830741740722684 | 15971.1041405269 | 15792.96699694 | 5.54921202870009 |
| Sham-5 | 0.98340587056082 | 16760.0214158239 | 16555.6961411998 | 7.67812356247128 |
| Sham-6 | 0.953140379384947 | 15289.9103690685 | 15227.0141652022 | 6.35289519328904 |
| MIRI-1 | 0.933731498298403 | 16349.3333333333 | 16350.6614469138 | 6.66625151246043 |
| MIRI-2 | 0.922674328474855 | 15787.2368908081 | 15622.0828418022 | 5.66928278952568 |
| MIRI-3 | 0.958178408181617 | 21147.5844968704 | 20838.4890833011 | 7.08085080096136 |
| MIRI-4 | 0.939451213274466 | 17415.4827018121 | 17308.5402824324 | 6.4157015456112 |
| MIRI-5 | 0.945201368082962 | 19759.74226254 | 19569.8965708559 | 6.63647541993347 |
| MIRI-6 | 0.967555338798114 | 20337.0823466092 | 20058.8726790258 | 7.16991003145702 |
| WLWDS-11-1 | 0.978847127926987 | 19989.2017625231 | 19878.919402659 | 7.50176884444033 |
| WLWDS-11-2 | 0.96581685299075 | 17734.0982194141 | 17498.9455704129 | 7.22467607038524 |
| WLWDS-11-3 | 0.963391971287413 | 16313.1008849557 | 16069.6515478094 | 6.81597728977143 |
| WLWDS-11-4 | 0.961538774828336 | 15674.6853932584 | 15579.4842623589 | 6.89937117964013 |
| WLWDS-11-5 | 0.974252720174181 | 17624.9828602035 | 17495.0567685602 | 7.18429300404638 |
| WLWDS-11-6 | 0.980198592199404 | 15721.0005810575 | 15673.1077807161 | 7.7297584864366 |
