# Supplementary figures and images for: Mongolian medicine Wulanwendusu-11 alleviates myocardial ischemia-reperfusion injury by modulating the intestinal microbiota and associated metabolic pathways
Source: Front Microbiol. 2026 Jan 9;16:1693472. doi: 10.3389/fmicb.2025.1693472 (PMC12828672; doi:10.3389/fmicb.2025.1693472)

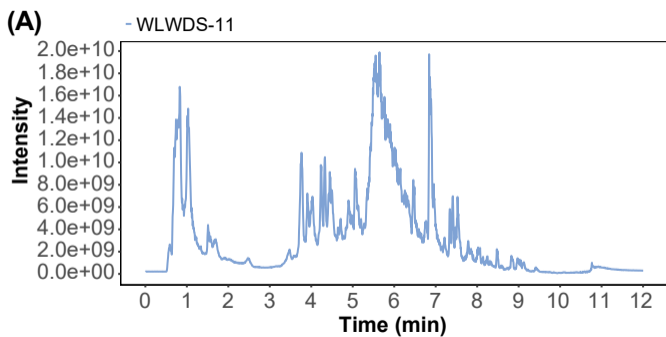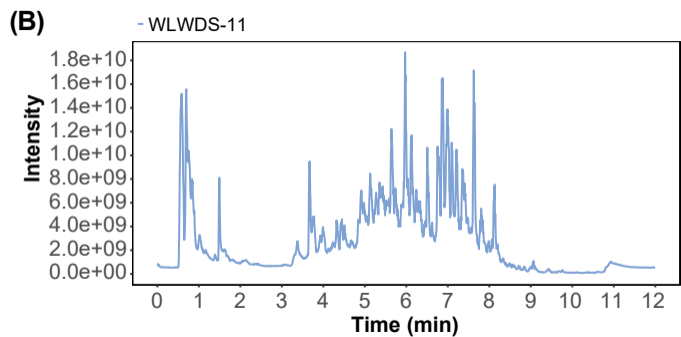

Supplement: Supplementary file 11 [file Image_1.pdf]

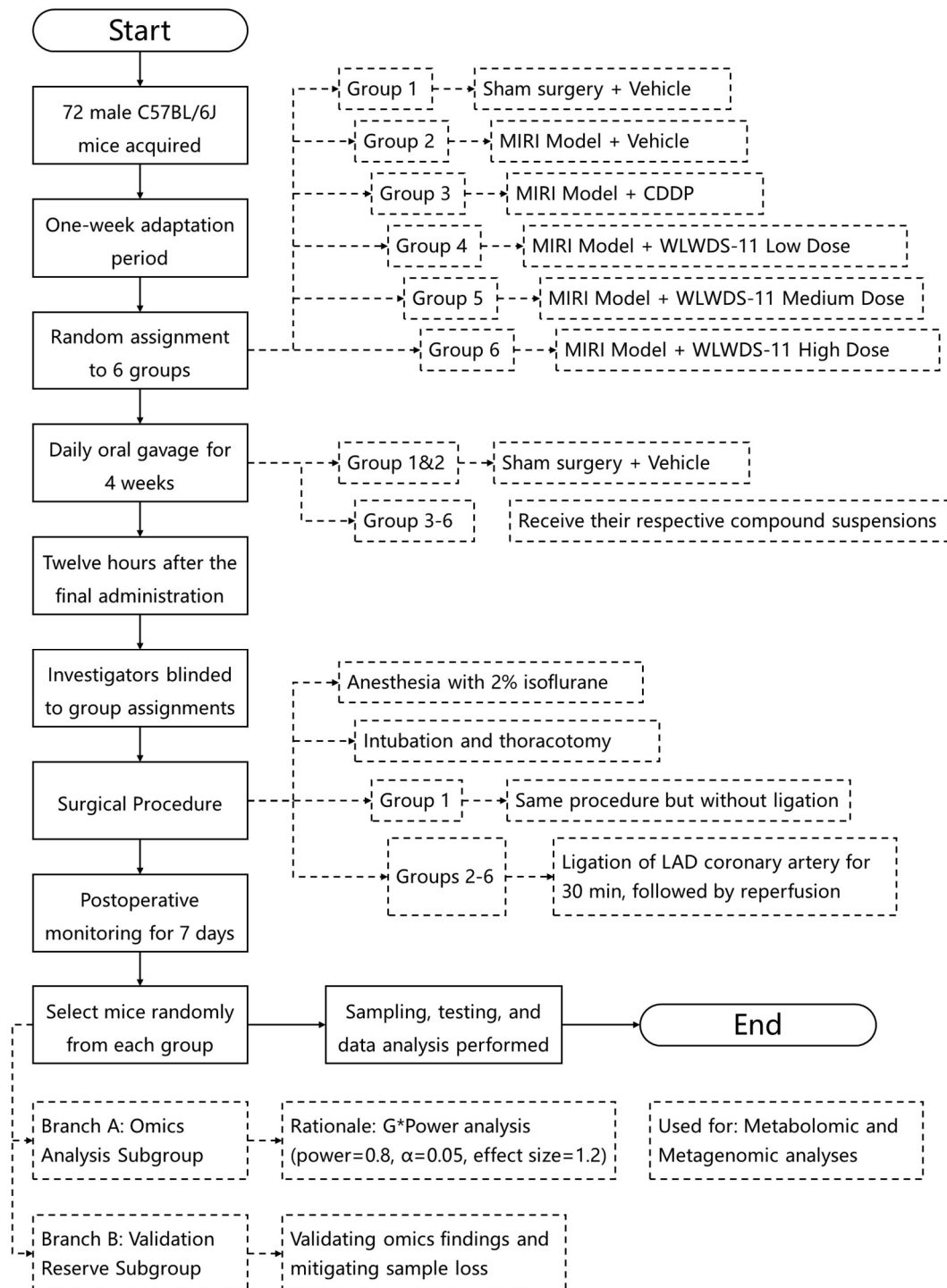

Supplement: Supplementary file 12 [file Image_2.pdf]

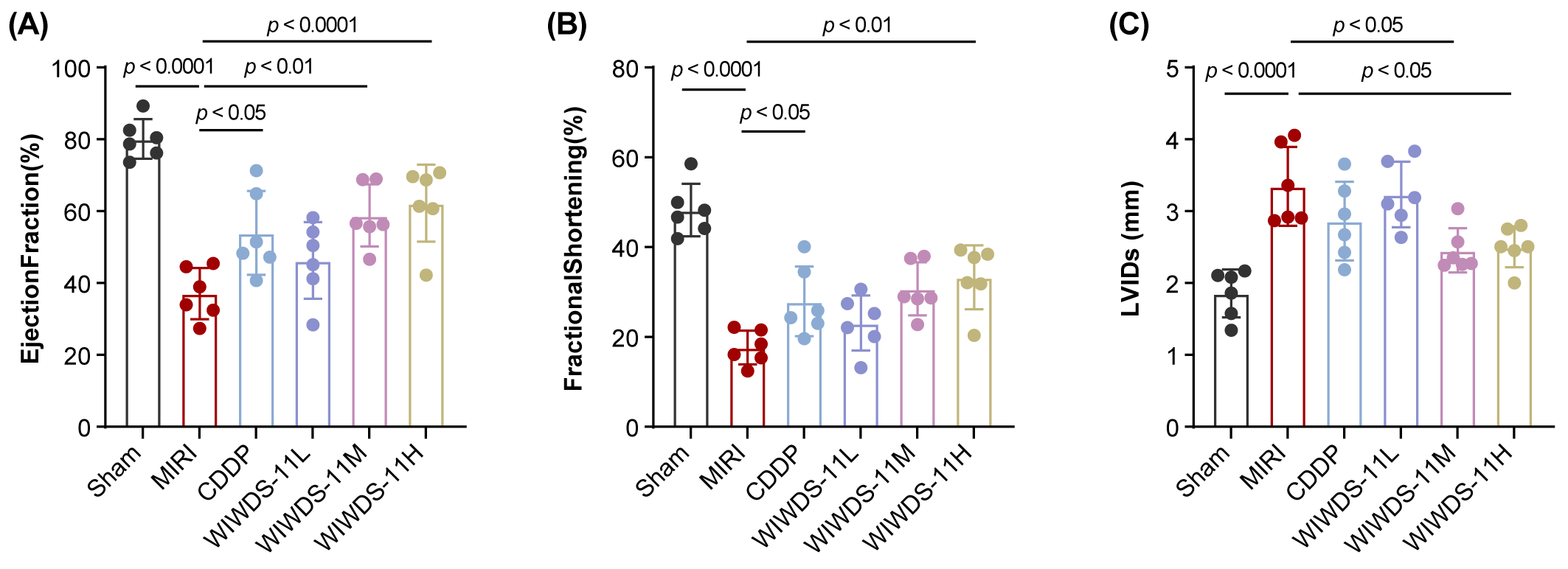

Supplement: Supplementary file 13 [file Image_3.tif]

Intercepts:  $R^2Y(\text{cum}) = (0, 0.91)$ ,  $Q^2(\text{cum}) = (0, -0.68)$

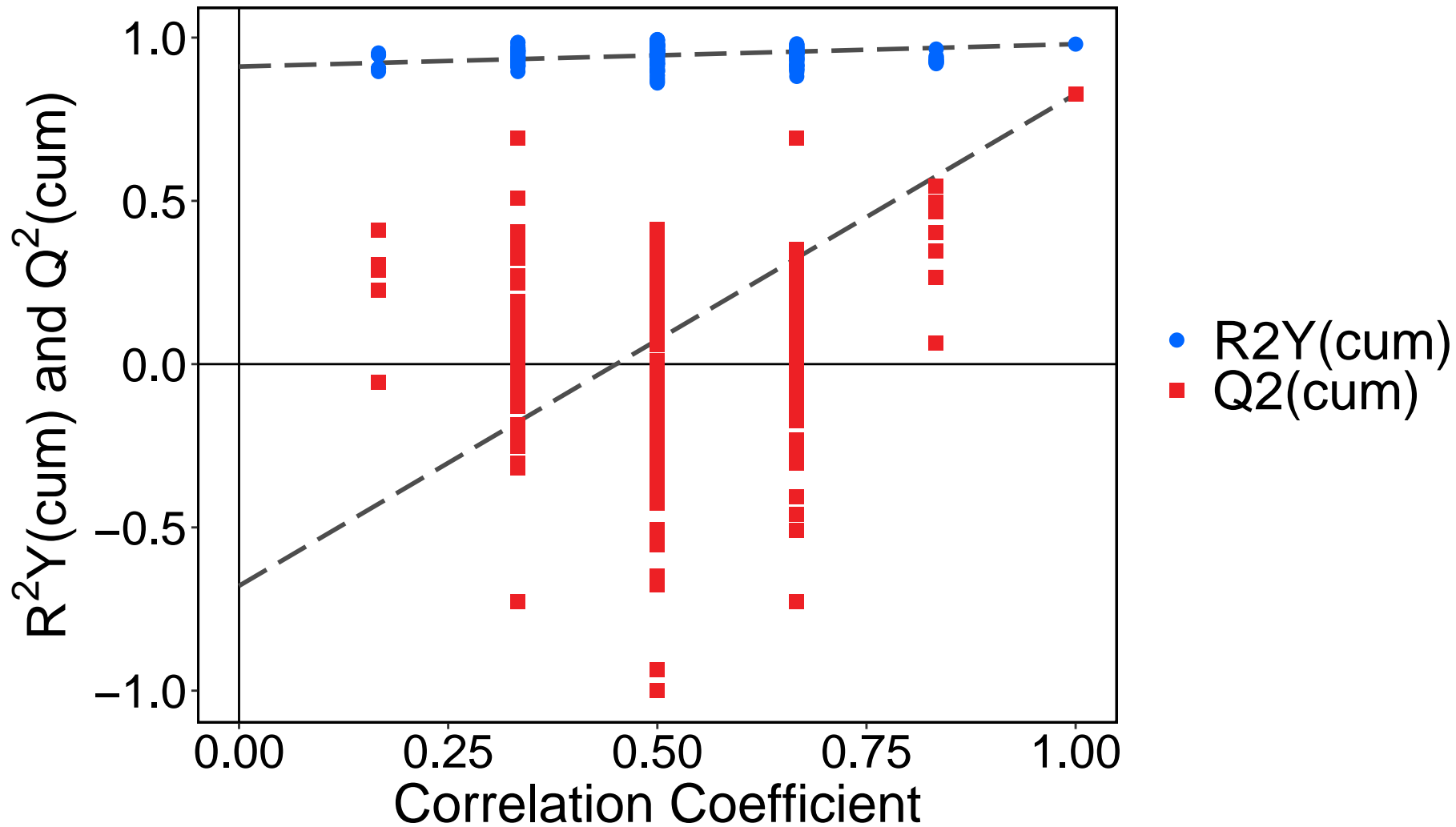

Supplement: Supplementary file 14 [file Image_4.pdf]

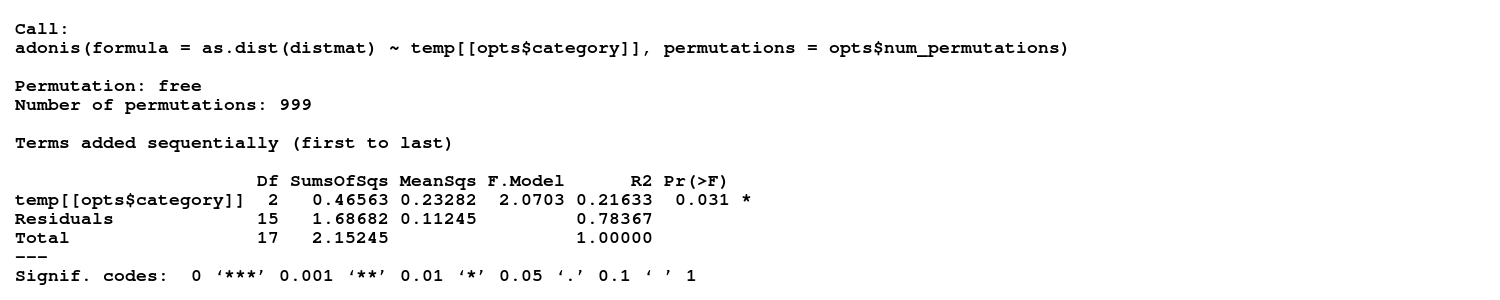

Supplement: Supplementary file 15 [file Image_5.png]
